# Important innate differences in determining symbiotic responsiveness in host and non-hosts of arbuscular mycorrhiza

**DOI:** 10.1038/s41598-021-93626-6

**Published:** 2021-07-14

**Authors:** Shalini Vasan, Divya Srivastava, David Cahill, Pushplata Prasad Singh, Alok Adholeya

**Affiliations:** 1 TERI-Deakin Nanobiotechnology Centre, Sustainable Agriculture Division, The Energy and Resources Institute (TERI), Gurugram, Haryana India; 2 School of Life and Environmental Sciences, Deakin University, Waurn Ponds Campus, Geelong, VIC Australia

**Keywords:** Computational biology and bioinformatics, Evolution, Genetics, Molecular biology

## Abstract

Genetic components that regulate arbuscular mycorrhizal (AM) interactions in hosts and non-hosts are not completely known. Comparative transcriptomic analysis was combined with phylogenetic studies to identify the factors that distinguish AM host from non-host. Mycorrhized host, non-mycorrhized host and non-host cultivars of tomato (*Solanum lycopersicum*) were subjected to RNA seq analysis. The top 10 differentially expressed genes were subjected to extensive in silico phylogenetic analysis along with 10 more candidate genes that have been previously reported for AM-plant interactions. Seven distantly related hosts and four non-hosts were selected to identify structural differences in selected gene/protein candidates. The screened genes/proteins were subjected to MEME, CODEML and DIVERGE analysis to identify evolutionary patterns that differentiate hosts from non-hosts. Based on the results, candidate genes were categorized as highly influenced (SYMRK and CCaMK), moderately influenced and minimally influenced by evolutionary constraints. We propose that the amino acid and nucleotide changes specific to non-hosts are likely to correspond to aberrations in functionality towards AM symbiosis. This study paves way for future research aimed at understanding innate differences in genetic make-up of AM hosts and non-hosts, in addition to the theory of gene losses from the “AM-symbiotic toolkit”.

## Introduction

Arbuscular mycorrhizal (AM) fungi colonize the roots of the majority of land plants and facilitate in uptake of essential minerals from the soil by extending their hyphal network to reach areas otherwise inaccessible to the host plant roots^1^. Mycorrhizae also increase host plant resistance to pathogens and tolerance to abiotic and biotic stress^2,3^. In return, the plant host supplies the fungal partner with essential carbon sources such as hexoses^4,5^ and lipids^6–8^ to facilitate AM fungal growth and reproduction.

Plant species that undergo AM symbiosis are called AM-hosts/AM-competent species. The remaining AM non-hosts either form associations with other types of mycorrhiza or in the case of non-mycorrhizal species do not form any mycorrhizal associations. Another group of plant species are weakly mycorrhizal, such as carnivorous plants and those species that form cluster roots^9–11^, which can switch from being an AM-host to non-host based on the availability of alternative nutrient sources. Early mycorrhizal research, aimed at understanding the inability of non-hosts to undergo AM symbiosis, was largely divided into two theories. The first theory proposed secretion of inhibitory compounds, such as glucosinolates, by the non-hosts to inhibit colonization by AM fungi^12^. While, the other theory proposed the absence of AM fungi growth stimulating compounds in non-hosts rather than inhibitory compound secretions^13^. The latter hypothesis was supported by another study which confirmed the presence of non-host does not influence AM spore germination and proliferation while the establishment of symbiosis requires host signalling stimulus that the non-host possibly lacks^14^.

Comparative transcriptomics between mycorrhized and non-mycorrhized host plants as well as between hosts and non-hosts have enhanced the understanding of AM symbiotic events to a large extent^15–17^. Reverse genetic approaches have been instrumental in validation of the gene expression changes and to decipher the signalling cascades involved in AM symbiosis^18–20^. Exhaustive in silico analysis have further enabled identification of fungal and plant targets involved in governing the symbiotic functioning^21–23^, provided insights into the common symbiotic signalling pathway (CSSP)^24,25^ and allowed large-scale analysis of gene loss and gene conservation in distantly related non-hosts and hosts to establish mycorrhizal symbiosis^11,18,26,27^. The novel findings led to a series of further investigations wherein the concept of ‘independent losses’ of AM symbiosis-specific genes in non-hosts came into consideration^28,29^. This was proposed to be the reason for the inability of non-hosts to form AM associations.

AM symbiosis is estimated to be a 400-million-year-old mutualism^30^ and the term “mutualism sustenance” is used to describe the ability of plants to form AM symbiosis as a function of the rate of evolution in terms of ‘events per million years’^9^. These ‘events’ are the evolutionary constraints that determine the strength of a symbiotic association and its fate in plant species that results in them being a strong host, non-host or a weakly mycorrhizal host^9^. Studies have confirmed that a core set of genes are conserved in AM-hosts^18,26,29^ to maintain the ability of these plant lineages to form AM symbioses while non-hosts undergo gene loss that leads to an inability to form mutualistic interactions with AM fungi.

This study aims to understand evolutionary relationships that differentiate between the AM host and non-host species. The focus of the present study is to understand the role of evolutionary pressure on protein structure and function of candidate genes that might influence AM-host and non-host status of different species (also compared amongst distantly related plant families). This study was focused on genes present in hosts that were specifically regulated in root in response to AM symbiosis. This was done by identifying differences in basal/non-mycorrhizal profiles of host and non-host compared to AM-inoculated host. The genes that had constituent expression were excluded from this study. Therefore, in this study the non-host (tomato cv. Grafter) has only been studied without mycorrhizal induction. Root organ cultures were used because the study was focused on locally and not systemically regulated genes.

We propose that the results are crucial to decipher how selection pressure may determine the outcome of host/non-host associations with AM fungi. Two sets of genes were selected. The first set consisted of the top 10 AM symbiosis-specific gene hits from the comparative transcriptome analysis between host and non-host species of *S. lycopersicum* at false discovery rate (FDR) ≤ 0.005. The comparisons between mycorrhized and non-mycorrhized host and non-host has also been conducted in our lab in order to map the metabolites regulations and associated genes that may govern AM symbiosis. However, that investigation is a part of another manuscript and cannot be included in this manuscript.

In order to enrich the putative candidate gene-set another set comprising of genes previously reported and well-known for their roles in AM symbiotic process was included in the analysis. While the analysed genes were present in both hosts and non-hosts, evolutionary constraints were shown to affect their cognate protein amino acid sequences and gene nucleotide sites which are likely to influence their respective function in AM-plant interactions. This study uses species-specific transcriptome changes towards AM symbiosis and identifies its commonalities throughout distantly related hosts and non-hosts via comparative phylogenetic analysis. Understanding the differences in genetic make-up native to host and non-hosts widens the horizon of mycorrhizal studies aimed at deciphering the AM-plant interactome.

## Materials and methods

### Selection of AM host and non-hosts

Four root culture lines (transformed with *Agrobacterium rhizogenes* LBA 9402) were screened for their potential to form symbiotic interactions with the AM fungus *Rhizophagus irregularis* DAOM 197198. These included a positive control which was a known AM host *Chicorium intybus*, negative control which was a known AM non-host *Brassica napus* cv. Canola and two test cultivars of *Solanum lycopersicum,* cv. Roma and cv. Grafter. All root lines were inoculated with 50 AM spores and screened for AM colonization post 10 weeks of inoculation via ink-vinegar staining method as described in Supplementary Method S1. The study complies with relevant institutional, national, and international guidelines and legislation.

### AM fungus and root culture preparation

Host and non-host cultivars of tomato (*S. lycopersicum*) were used. The host cultivar was tomato cv. Roma and non-host cultivar was tomato cv. Grafter (new Gippsland seeds and territorial seed company) an F1 grafted hybrid of tomato cv. Carmelo^31^. The in vitro root organ cultures (ROCs) for these plant lines, transformed using *Agrobacterium rhizogenes* LBA 9402^32^, were kindly provided by Prof John Hamill (Centre for Regional and Rural Futures, (CeRRF), Deakin University, Australia). *Rhizophagus irregularis* spores established as in vitro dual culture with *Daucus carota* (carrot) were obtained from the Centre for Mycorrhizal Culture Collection (CMCC), The Energy and Resources Institute (TERI), Gurugram, Haryana, India. All in vitro root cultures were maintained at 26 °C in the dark.

### Sample preparation

Root cultures without mycorrhizal inoculation (NMyc) were prepared using active root segments (5–7 cm in length with 2–3 lateral root tips) excised from the stock culture (~ 25 days old), using a scalpel, within a laminar flow cabinet and transferred using sterile forceps to a Petri dish (Tarsons, India; 90 × 15 mm) containing M media^33^. The plates were then labelled, sealed with parafilm and incubated at 26 °C in the dark.

For AM-inoculated host cultures, 8-week-old in vitro* R. irregularis* spores were harvested from the carrot-AM dual culture^34^. The spores were washed in 10 mM sodium citrate buffer^35^ to remove adhering media and then washed with sterile distilled water. Fifty spores were placed in the centre of a sterile M media Petri plate (150 mm in diameter and depth of 15 mm) and incubated at 26 °C for 24 h in the dark. One or two root segments with lateral root tips were excised from non-mycorrhized tomato Roma (host) culture (~ 25 days old) and placed 2 cm from the AM fungal spores. Plates were sealed and incubated at 26 °C in dark. Five replicates for each host line were prepared and the experiment was repeated.

The roots were harvested after 22 days, washed in 10 mM sodium citrate buffer^35^ and then with sterile distilled water through a sieve (52 British Standard Sieve, Industrial Wire Netting Co., New Delhi, India). Washed roots were gently blotted dry and then 150 mg of root biomass was apportioned to each replicate per sample. The samples were then frozen in liquid nitrogen and either subjected to RNA isolation immediately or stored at − 80 °C until further use (Supplementary Table S1).

### RNA extraction and purification

Frozen root tissue was ground in liquid nitrogen and subjected to RNA isolation using the Ambion Purelink RNA mini kit (Invitrogen, California, USA) followed by RNA purification using the DNase 1 kit (Sigma Aldrich, St. Louis, Missouri, United States) as per the manufacturer’s instruction(s). Two biological replicates were used for each sample. RNA quality was evaluated using the Nanodrop 2000c (Thermo Scientific, Massachusetts, USA) and 1% Agarose gel electrophoresis in TBE buffer. Details of RNA seq analysis and quality control are mentioned in Supplementary Method S2 and S3 respectively). RNA seq results were validated via qRT-PCR. The Ambion Purelink RNA extraction and DNase 1 purification protocols were also used to prepare samples for qRT-PCR validation. The details of qRT-PCR validation are provided in Supplementary Method S4.

### Selection of candidate genes for phylogenetic analysis

**Figure 1:**
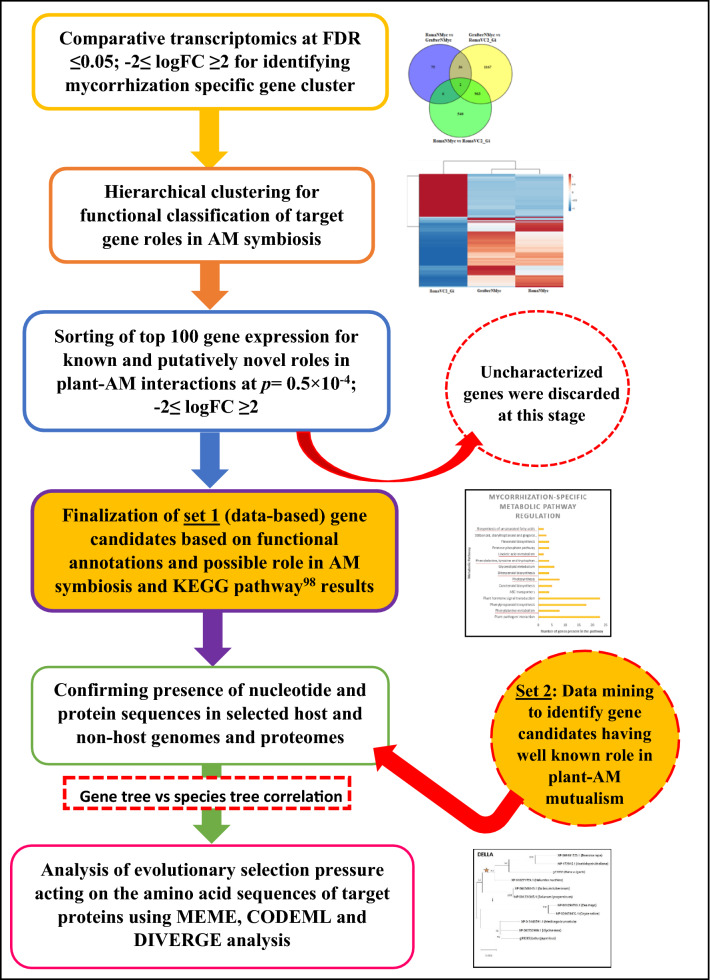
Schematic representation of workflow. This figure provides an overview of the phylogenetic analysis workflow adapted in this study. The images accompanying methods are representative examples of results obtained. These are enlarged/explained in “Results” section.

Two sets of genes were explored in this study. Set 1 consisted of “putative-novel candidate genes” identified from RNA seq analysis carried out in this study that compared a non-host cultivar (Grafter) of tomato with a host cultivar (Roma) under AM inoculation and AM-inoculation free states. Putative novel candidates are those genes that have not been well studied with respect to AM symbiosis before but their expression profiles indicated a role in AM-plant interactions in our study. Set 2 consisted of the previously reported “known candidate genes” that have established roles in the regulation of AM symbiosis (as mentioned in Tables 1 and 2).This set included candidate gene sequences from seven host and four non-host genomes that were also used in previously reported phylogenomic studies on plant-AM interactions^18,28^. This study was aimed at understanding the conservation or diversification in genes, which could manifest in functional alteration in function due to evolutionary factors such as selection pressure^11,26^ and/or speed of evolution^36^. For this purpose, the proteomes of leguminous as well as non-leguminous families that have both dicot and monocot species were selected to enable wider applicability of results (Supplementary Table S2). Confirmation of candidate gene/proteins in selected proteomes was done as mentioned in Supplementary Method S5. The details of conserved domain analysis (CDD) are mentioned in Supplementary Method S6. An overview of the workflow used for phylogenetics analysis is provided in Fig. 1.

**Table 1:** List of putative novel gene candidates (set 1) used for phylogenetics and details of symbiotic-role.

Gene name (locus information)	Status in mycorrhized host	AM symbiosis associated role (known/ unknown)
*6-Phosphogluconate dehydrogenase decarboxylating* (**12:62087150–62088641**)	Up-regulated	Unknown
*AAA-ATPase At4g25835-like* (**1:81070083–81071866**)	Down-regulated	Unknown
*Ankyrin repeat PH and SEC7 domain containing protein secG-like*^a^ (**9:66080841–66082224**)	Down-regulated	C terminus domain of VAPYRIN (protein that is a part of the common symbiotic signalling pathway (CSSP))^76^
*GATA transcription factor* (**1:97046157–97047704**)	Down-regulated	Unknown
*Pathogenesis-related genes transcriptional activator PTI6* (**6:48363699–48364446**)	Down-regulated	Unknown
*Probable LRR receptor-like serine/threonine-protein kinase At1g74360* (**3:65257764–65261637**)	Down-regulated	Ser/thr kinase is involved in peri-arbuscular membrane formation and ser/thr domain is present in signalling genes such as CCaMK^77^. However, LRR receptor ser/thr kinase function specific to AM symbiosis is still unknown
*β-expansin* (**3:54655131–54656997**)	Up-regulated	Arbuscule development^68^
*Putative calcium-binding protein CML45-like* (**2:50279342–50279888**)	Down-regulated	Unknown
Scarecrow-like protein 5; scarecrow-like protein 13* (**5:64111514–64112957**)	Down-regulated	Scarecrow proteins are part of the GRAS transcription factors regulating extent of AM symbiosis in host roots^78^
*S*-*adenosyl-L-methionine: salicylic acid carboxyl methyltransferase* (**9:70802563–70805153**)	Up-regulated	Unknown

^a^Deviations from expected expression status observed in previously published literatures.

**Table 2:** List and symbiotic-role of known gene candidates (set 2) used for phylogenetics.

Gene name	Role in AM symbiosis	References
*LYK10*	Lysin motif receptor like kinases, perceiving Myc COs and Myc LCOs pre-contact and required for AM penetration of plant roots	^22^
*LYK12*	Required for colonization by AM fungus; belongs to both Solanaceae and Leguminosae species clade	^69^
*CCaMK*	Initiator of calcium signalling cascade in hosts; works in accordance with CYCLOPS and DELLA to activate RAM1 required for arbuscule development	^56,57^
*DELLA*	Antagonism to GA; involved in regulating the extent of AM symbiosis having synergistic function with CYCLOPS-CCaMK to activate RAM1	^16,64^
*CASTOR*	Twin ion channels; required for AM colonization of roots	^74^
*POLLUX*		
*HMGR-CoA*^a^	Initiates the calcium spiking needed for activation of downstream transcriptional responses to AM symbiosis	^19,62^
*CCD7*^a^	Involved in strigolactone biosynthesis important for host perception by AM fungus	^79,80^
*CCD8*		
*SYMRK*	Symbiotic receptor-like kinase; required for early signalling events and transduction of signals to activate downstream cascades	^54,55^
*CNGC15*	Required for nuclear localised symbiotic calcium oscillations	^81^
*CYCLOPS/IPD3*^b^	Required for arbuscule initiation; part of the CCaMK-DELLA-CYCLOPS complex activating many transcription factors required for symbiotic association including RAM1	^11,57^

^a^Literature candidates found in transcriptomics data.

^b^Absent in non-host proteomes (not used in further analysis).

### Maximum likelihood gene tree and species tree via MEGAX

Gene trees help trace the evolutionary changes that occur in the ‘gene of interest’ in selected species. MEGAX^37^ (Molecular Evolutionary Genetics Analysis) was used to construct the Maximum Likelihood (ML) tree at a bootstrap value of 1000, using the aligned protein fasta file as input. The output was obtained in .png and .nwk formats. Species tree was prepared for the 11 selected proteomes using 59 nuclear conserved genes that have been previously recommended for phylogenetic analysis in angiosperms^38^. Protein sequences were fetched from NCBI. HAL tool^39^ was used for MSA and clustering of proteins based on orthologous groups (http://sourceforge.net/projects/bio-hal/). Any poorly aligned sequences and gaps were concatenated using FASconCAT (https://www.zfmk.de/en/research/research-centres-and-groups/fasconcat) to merge all the protein sequences from all species into one super alignment file and saved in fasta format. This protein file was used to construct the species ML tree using FastTree2^40^ set at Dayhoff amino acid substitution model and 1000 bootstrap replications. The output was saved in.nwk and .png formats. *Neurospora crassa* was used as an outgroup species.

### MEME to identify motifs that contribute to gene function

Multiple EM (Expectation Maximization) for Motif Elicitation (MEME) tool^41^ (http://memesuite.org/) was used to identify the motifs contributing towards gene functionality in comparison to conserved motifs and tracing any patterns corresponding to selection pressure. Parameters adjusted for MEME analysis included: number of repetitions (zero or one occurrence per sequence (zoops)), number of motifs (5 best motif hits) and width of sequence set min/max (5–15 amino acids). The protein fasta file aligned and concatenated was used as input to analyse motif deviations in conserved regions while, the raw sequences obtained from NCBI were used to analyse any host/ non-host specific patterns in non-conserved motifs that might be contributing to protein structure and function.

### Synonymous versus non-synonymous mutations

Non-synonymous (dN) mutation is one where a nucleotide substitution in the codon leads to change in amino acid sequence that reflects on protein functionality. On the other hand, synonymous (dS) mutation is when this nucleotide change does not alter the amino acid the codon translates to. The ratio of mutation is given by ω = dN/dS^42^. If ω > 1, it depicts positive selection or “adaptive evolution” while ω = 1 suggests neutral evolution and ω < 1 represents purifying selection (this might lead of independent gene loss or loss-of-gene function for a particular phenomenon, in this case, AM symbiosis). Selection pressure contributes to shaping a protein’s biological function to correspond with changing environmental requirements. CODEML module of the PAML (Phylogenetics Analysis using Maximum Likelihood) tool v4.9 is extensively used to trace evolutionary changes in codons. In addition, this tool is also used to estimate likelihood ratio tests (LRTs) to compare various substitution models that best fit the gene(s) of interest with insights on divergence times and type of mutations (dN/dS)^43^. Site and branch models were used to trace the nucleotide site and gene tree branch positions that experienced evolutionary pressures respectively, to re-establish functional roles. Models M2a and M8 are used for detecting positive selection, M3 suggests variable selection pressure while models M1a and M7 represent the neutral hypothesis for site models^44^. For the current study branch models, each newick gene tree was altered to select the gene tree branches to be analysed for selection pressure (inserted with #) and were run at ω fixed value 1 and 0 individually. Nucleotide gene sequences were prepared so that the total number of bases was rounded off to the nearest multiple of 3 so as to serve as input for CODEML. This was compared to the respective (protein) gene tree obtained in .nwk format previously. Both terminal runs and output files were compiled.

### Functional analysis using DIVERGE

The DIVERGE tool^45^ (DetectIng Variability in Evolutionary Rates among GEnes) allows two kinds of functional divergence analysis: type I divergence corresponds to the constraint exerted on protein residues as a result of gene duplication at particular sites amongst paralogous clades by evolutionary speed/rate, type II divergence represents occurrences of mutation due to early events that lead to gene duplication altering amino acid sequence and thus, the protein functionality which becomes conserved over time^46^. Protein fasta files were used as input for DIVERGE tool version 3.0 along with the Newick gene tree to form clusters of interest, in this study, host versus non-hosts. The output was tabulated and interpreted to analyse the effect of environmental conditions on gene functions.

## Results

### Confirming host and non-host status of tomato cultivars

**Figure 2:**
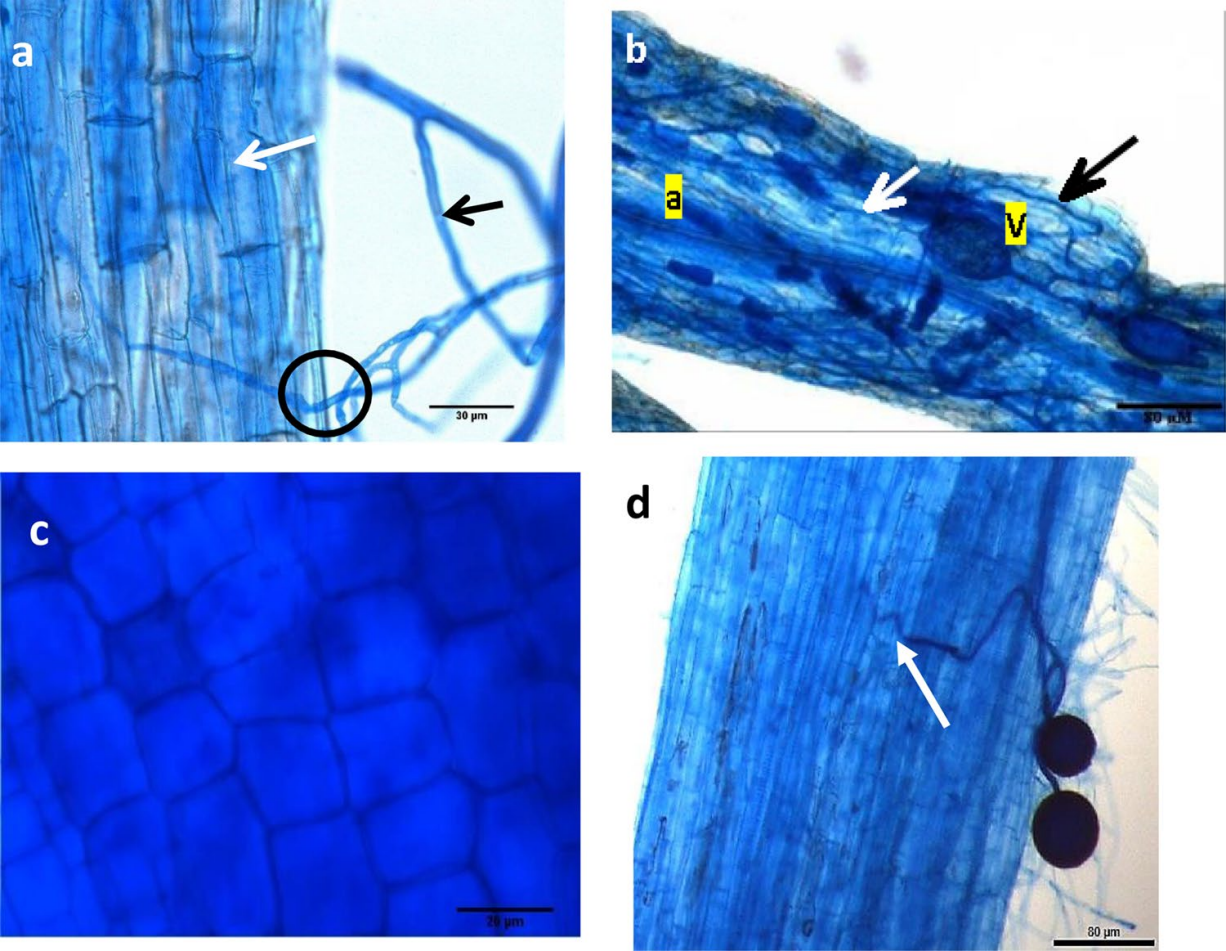
Screening of root cultures for host and non-host potential with respect to the AM symbiosis. (**a**) Possible point of entry of AM fungus into the root segment (appressorium; depicted by black circle) might be representative of initial contact enabling AM fungal penetration into the roots; (**b**) Established host culture showing well developed vesicles (V) and collapsing arbuscules (**a**) connected via hyphal network (white arrow shows the host cells while the black arrow shows AM fungal hypha); (**c**) Tomato cv. Grafter root cortical cells devoid of any AM fungal structures confirm their inability to sustain the symbiosis, (**b**) root segments of tomato cv. Grafter that has possible signs of mycorrhizal entry (white arrows) but no markers of successful symbiosis such as well-developed hyphal network, arbuscules or vesicles.

The AM colonization percentage for *Solanum lycopersicum* cv. Roma was ~ 62.80% and for *Cichorium intybus* it was ~ 61.85% (representative example shown in Fig. 2). Negative control, *Brassica napus* cv. Canola and test cultivar *Solanum lycopersicum* cv. Grafter did not show any signs of colonization with AM fungus *Rhizophagus irregularis* hence, was selected as the AM non-host for this study. Also, tomato cv. Grafter provides genetically similar background to tomato cv. Roma so it was used as a “surrogate non-host” of AM fungus *R. irregularis*.

### Comparative transcriptomics between AM host and non-host species of *S. lycopersicum* (SRA BioProject ID PRJNA608804)

Two biological replicates of AM-inoculated host (RomaVC2_Gi), non-inoculated host (RomaNMyc) and non-inoculated non-host (GrafterNMyc) were subjected to RNA seq analysis (see Supplementary table S1). A total of 1385 genes were up-regulated and 1381 genes down-regulated in the AM host RomaVC2_Gi for 21 days in comparison with GrafterNMyc while 809 and 1180 genes were up- and down-regulated respectively in comparison to RomaNMyc at FDR ≤ 0.05; -2 ≤ log fold change ≥ 2. Reads were mapped as per the FPKM (fragments per kilobase of transcript per million mapped reads) values and the differential expression for comparative sets plotted (Supplementary Fig. S1). RNA seq results were partially validated via qRT-PCR (Supplementary Fig. S2). Randomly selected ten genes were identified to show differential expression between host and non-host at FDR ≤ 0.005 in comparative transcriptome analysis. The genes showed same trend of expression (up- and down- regulation) as was observed in RNA seq. Three biological replicates (with three technical replicates for each) were used in qRT-PCR analysis. Absence of DNA contamination in samples used for qRT-PCR analysis was confirmed via Nanodrop quantification (Nanodrop 2000c spectrophotometer, Thermo Fischer Scientific, Massachusetts, United States) and 1% agarose gel electrophoresis as shown in Supplementary Fig. S3. Nanodrop values for all the RNA samples were between 2.1 and 2.2, confirming their purity.

### Functional characterization of novel candidates identified in RNA seq analysis

**Figure 3:**
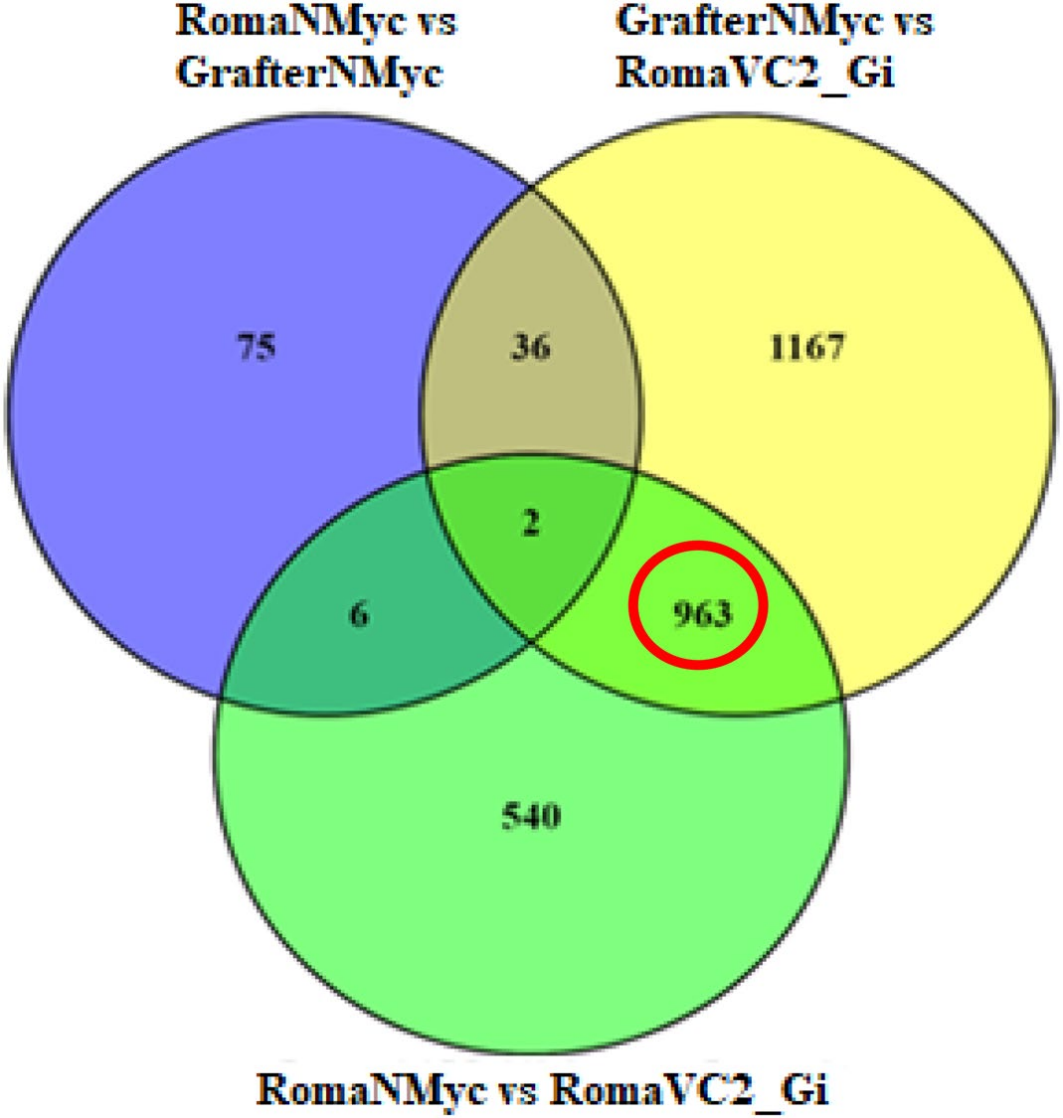
Comparison of the number of differentially expressed genes (DEGs) (up and down-regulated) for the non-inoculated host and non-host with AM-inoculated host. Venn diagram represents DEG profiles at FDR ≤ 0.05 and − 2 ≤ log fold change ≥ 2. Genes exclusively expressed in the basal root transcriptome (without any AM inoculation) is represented by the non-overlapping area of the blue circle (RomaNMyc vs GrafterNMyc). Yellow circle (excluding overlap area) represents DEGs in AM-inoculated host (RomaVC2_Gi) compared to non-inoculated non-host (GrafterNMyc). Non-overlapping region of green circle represents DEGs in AM-inoculated host (RomaVC2_Gi) compared to non-inoculated host (RomaNMyc). The 963 genes constituting the overlapping region (light green) in these two sets, are DEGs specific to AM-inoculation in hosts (up- and down-regulation) as these do not overlap with basal root profiles (blue circle). These genes were selected for further characterization.

The Venn diagram (Fig. 3) compares three sets of DEG profiles: I. transcriptome difference between non-inoculated host (RomaNMyc) and non-host (GrafterNMyc) categorized as ‘basal expression profiles’, II. transcriptome differences in GrafterNMyc and AM-inoculated host (RomaVC2_Gi) while III. compares RomaNMyc with RomaVC2_Gi. Nine hundred and sixty-three genes were found to be differentially regulated in RomaVC2_Gi when compared with both RomaNMyc and GrafterNMyc. This 963 gene cluster (red circle; Fig. 3) was devoid of the genes expressed in the basal profiles of host and non-hosts. Hence, this cluster was used to identify plant genes specifically regulated in response to AM colonization. These genes were grouped into two categories, i) known candidate genes and ii) transcriptome-data based (putatively novel) candidate genes.

**Figure 4:**
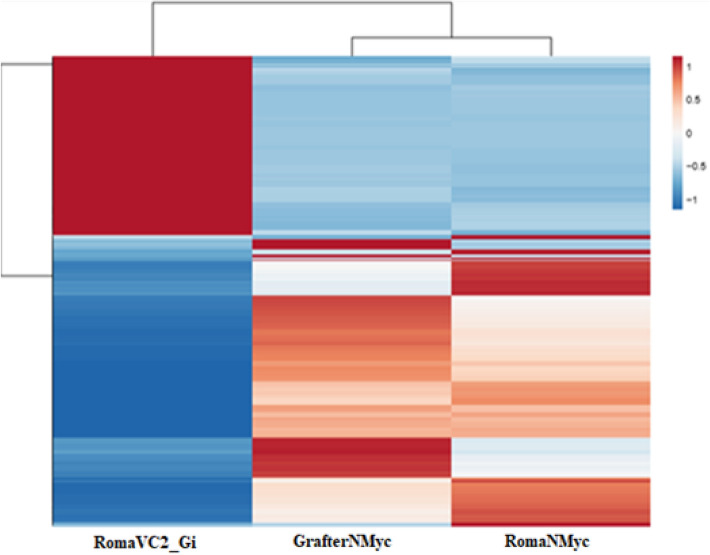
Heatmap to visualize hierarchical clustering of AM symbiosis-specific genes (up- and down-regulated) between AM-inoculated host and non-inoculated host and non-host. This figure represents heatmap for 963 mycorrhization-specific genes obtained by comparative RNA seq analysis of AM-inoculated host (RomaVC2_Gi) with non-inoculated host (RomaNMyc) and non-host (GrafterNMyc) clearly depict a subset of genes that are strongly upregulated (red) and downregulated (blue) in mycorrhized host root (RomaVC2_Gi) when compared with both non- inoculated host (RomaNMyc) as well as non- host (GrafterNMyc).

Hierarchical clustering (Fig. 4), constructed via ClustVis tool^47^, was used for functional classification for the 963 mycorrhiza-specific genes demonstrated in Fig. 3. Out of the 963 genes, 368 were up-regulated (red cluster, Fig. 4) while 595 genes (blue cluster, Fig. 4) were down-regulated in RomaVC2_Gi compared to RomaNMyc and GrafterNMyc. These included genes that encoded 526 uncharacterized proteins, 51 putative ovule proteins, 12 ring type E3- ubiquitin transferase and 9 xyloglucan endotransglycosylase in addition to several multi-loci duplicates of genes that encoded serine/threonine kinases, an F-box kelch protein, peroxidases and mitogen-activated protein kinase amongst others. Finally, 30 up-and 33 down-regulated gene targets were further categorized at *p* value = 0.00005 based on their gene regulation in response to AM-inoculated and non-inoculated conditions.

These candidate genes were simultaneously subjected to GO enrichment analysis (Supplementary Fig. S4) using KOBAS (KEGG Orthology Based Annotation System) 3.0.0 tool^48–50^. Gene coordinates from 10 out of the top 15 KEGG (Kyoto Encyclopedia of Gene and Genomes; https://www.genome.jp/kegg/) pathway hits were selected for further phylogenetics analysis based on their functional relevance to AM symbiosis and termed “putative novel candidate genes”, which constituted set 1. The ten gene targets selected for qRT-PCR validation studies were also part of these KEGG pathways differentially regulated in response to AM inoculation.

### Functional significance of gene targets selected for phylogenetic analysis

Tables 1 and 2 represent the two sets of gene targets used for this study, putative novel candidate genes (set 1) and known candidate genes (set 2), respectively. Locus information listed in Table 1 corresponds to RNA seq data deposited in SRA bioproject ID PRJNA608804**.** Supplementary Table S3 and S4 represent the details of CDD^51^ output obtained for all the selected genes (host and non-hosts) to identify the conserved domains and confirm their presence or absence in selected proteomes.

### Tracing evolutionary history using species and gene trees

**Figure 5:**
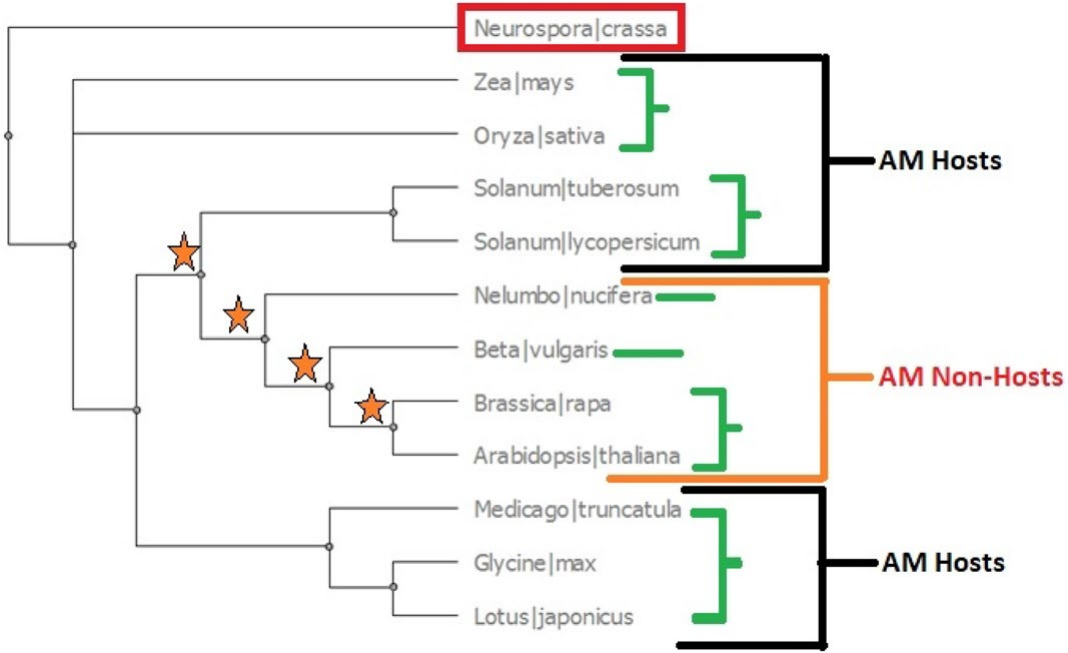
Species tree for selected host and non-host genomes. Green brackets represent host and non-host family clusters. Black brackets and Orange brackets are representative of Host and non-host species respectively. Red box represents the outgroup while orange asterisk signifies the event that lead to non-host lineage emergence. All non-host species emerge from one speciation event and further diverge depending on the environmental conditions. This tree served as a blue print to understand whether a gene is affected by plant speciation or has an independent process of experiencing evolutionary constraints when correlated with the gene trees.

For phylogenetic analysis, the species tree (Fig. 5) was constructed using the nuclear genes conserved in angiosperms^18^. Forty-five of the recommended 59 genes aligned with the selected genomes and were used to construct the ML tree using *Neurospora crassa* as the outgroup species. The species tree was used to estimate the relatedness between the host and non-host species and to correlate the evolutionary events with the gene tree of the respective symbiotic gene targets. In terms of relatedness, there was one main event that led to emergence of non-host lineage(s), which was followed by many more that further shaped the non-host distribution.

The gene trees were divided into three categories based on the total number of branches that segregated non-hosts. Supplementary Fig. S5-S8 depicts the representative gene trees for each category. Category 1 (Supplementary Fig. S5) consisted of gene trees that were exactly in line with the species tree and suggested that a single evolutionary event separated non-hosts from the host. This category included *DELLA* and *SYMRK*. Category 2 (Supplementary Fig. S6) included genes where the non-hosts diverged into two evolutionary branches, The genes belonging to category 2 included *LYK10*, *LYK12*, *HMGR-CoA*, *POLLUX*, *AAA-ATPase*, *ß-expansin*, *CCD7*, *CCaMK*, *calcium binding protein (CML45-like)* and *LRR receptor-like Ser/Thr protein kinase*. Category 3 (Supplementary Fig. S7) consisted of the gene trees that did not show any host/non-host specific clustering for the respective gene targets. This category included *CCD8*, *CNGC15*, *scarecrow-like protein*, *S-adenosyl-L-methionine*, *6-phosphogluconate dehydrogenase*. The final set of genes included *CASTOR*, *ankyrin repeat*, *PR genes transcriptional activator (PTI6)* and *GATA transcription factor* that did not show any signs of evolutionary pressure to distinguish AM hosts from non-hosts (Supplementary Fig. S8). Hence, these were not evaluated in further analysis. Similarly, as *CYCLOPS* was exclusively found in the selected host proteomes, it was also not included in downstream phylogenomic analysis. The divergent branches identified in gene trees (identified under category 1 and category 2; Supplementary Fig. S5 and S6) were used for branch-site models in CODEML analysis.

### MEME to identify differences in conserved motif sequences

The MEME tool^41^ was used for identification of motifs contributing to the biological function of a conserved gene sequence. Five motifs for each gene were predicted and screened for differences amongst the selected host and non-hosts.

Supplementary Fig. S9-S11 illustrates the best motifs predicted for all the symbiosis-related protein candidates. An amino acid change found in at least 3 out of 4 selected non-host species was highlighted. DELLA, SYMRK and calcium binding protein (CML45-like) were found to have motifs with amino acid changes specific to the four non-hosts (Supplementary Fig. S9). DELLA shows amino acid substitution specific to non-hosts from leucine (L) and isoleucine (I) to methionine (M). In SYMRK a substitution of proline (P) + phenylalanine (F) to glutamic acid (E) + tyrosine (Y) specific to the non-host was observed. While a shift from F to Y does not change the hydropathy or charge since both are non-charged hydrophobic amino acids, a shift from P to E is substitution by a negatively charged hydrophilic amino acid. Calcium binding protein (CML45-like) also depicted specific shift to lysine (K) in all non-hosts compared to a mixed amino acid pool for the same position in hosts (although this motif was not predicted in *O. sativa*).

Another predicted motif for CML45-like protein found in all hosts and non-hosts showed specific amino acid sequence changes to glutamine (Q) and serine (S) in 3 out of 4 non-hosts (excluding *Nelumbo*; result not shown). One of the predicted motifs in CCaMK also followed similar pattern of amino acid sequence changes. In CCaMK, positional as well as amino acid changes were evident between host versus at least 3 non-hosts species (*Arabidopsis*, *Brassica* and *Beta*; Supplementary Fig. S10). The positional shift occurs from 145th amino acid position to 112th in non-hosts (excluding *Nelumbo*). Amino acid shifts of lysine (K) and arginine (R) to valine (V) and aspartate (D) to serine (S) are all seen in these three non-hosts that signify loss of charge.

Similarly, the protein AAA-ATPase shifts from uncharged amino acids valine (V), serine (S) and alanine (A) to negatively charged glutamic acid (E) for non-host proteomes (excluding *Nelumbo*; Supplementary Fig. S10). It also represents a change in adjacent amino acid sequence from arginine (R) and cysteine (C) to lysine (K) wherein R and K are both positively charged hydrophilic amino acids. HMGR-CoA shows change in amino acid but not in the characteristic since change from aspartate (D) to glutamic acid (E) in non-hosts (excluding *Nelumbo*; Supplementary Fig. S10) are both hydrophilic and negatively charged while one of the predicted motifs for LYK-10 also showed changes of amino acid sequences specific to 3 of 4 non-hosts compared to hosts (result not shown). The remaining proteins did not have any remarkable differences between different plant species investigated in the study (Supplementary Fig. S11).

### CODEML to determine extent of selection pressure

CODEML tool^43^ enabled maximum likelihood (ML) estimates of evolutionary pressure acting on the nucleotide sequences that code for proteins (codons) of selected gene targets in comparison to multi-species gene tree (Supplementary Fig. S5-S8). Codons were analysed for synonymous and nonsynonymous mutations in specific sites via the “site model” by correlating ω values with the statistical models used (M0, M3, M1a, M2a, M7 and M8). On the other hand, branch-site models helped identify non-host tree branches having sites under evolutionary constraints; used as foreground branch, estimated via the f: ω2a and f: ω2b values in comparison to the remaining species (background branch).

LRT ratios are important to determine whether the statistical models favour positive selection or not. This is done by comparing null/neutral models with models that allow positive selections in codon sites; ω > 1^43^. Bayes Empirical Bayes (BEB) model assists identification of exact sites that are under selection based on the LRT values at *p* ≤ 0.05^43,44^. Supplementary dataset 2 represent the site and branch-site models respectively, used for set 1 and set 2 gene candidates. The ω values were used to understand status of genes with respect to rate of evolution.

Results indicated that, in terms of LRT ratios, most of the genes favoured variable selection by having the highest M0/M3 LRT ratios such as DELLA, CCD7, *scarecrow-like protein* and *6-phosphogluconate dehydrogenase* amongst many others. Mostly the ω value suggested a tendency towards positive selection in site models that is very well supported by BEB positively selected site predictions as in the case of *SYMRK*, *CNGC15*, *ß-expansin* and *LRR receptor-like Ser/Thr protein kinase*. Branch site models also showed very high selection values for the majority of the non-host foreground branches selected in Supplementary Fig. S5-S8. However, BEB sites were predicted for only a few of these gene candidates that include *CCD7*, *CNGC15*, *ß-expansin* and *scarecrow-like protein*.

Some representative examples include ω values for site and branch site CODEML models for SYMRK that favours non-synonymous mutation. This strongly suggests the influence of adaptive evolution in congruence with the change in amino acid pattern specific to non-host evolutionary branching(s); M0: ω = 1.59525, M2a: ω2 = 2.55037, M3: ω1 = 1.70007; ω2 = 3.29119, M8: ω = 2.51056 and; branch site model foreground ω = 13.54 (supplementary dataset 2). Similarly, the *CCaMK* CODEML site model supports variable selection (M0/M3 LRT) with BEB sites supporting positive selection that signifies the presence of non-synonymous mutation; M3: ω2 = 1.04856; M8: ω = 1.12687 (supplementary dataset 2). The branch-site model also supported this hypothesis with f:ω2a and f:ω2b values of 313.01 and 22.95 for selected non-host branches. Similarly, *DELLA* site models of CODEML also support the theory of adaptive evolution via positive selection; M2a: ω2 = 1.84500 and M8: ω = 1.67006 while AAA-ATPase site models; M3: ω2 = 1.71664 and M8: ω = 10.31970 support variable evolutionary pressure.

Another gene target, *6-phosphogluconate dehydrogenase*, showed CODEML site models that strongly support positive selection with M2a: ω2 = 38.18608. Moreover, the branch-site model evaluating branch 2 comprising of *Arabidopsis*, *Beta* and *Brassica* species having a f: ω2a and ω2b = 289.34160 and branch 1 comprising of *Nelumbo* having a f: ω2a and ω2b = 1.29720 signified that the protein residues are experiencing evolutionary constraints. LYK12 site model supports adaptive evolutionary constraint via M2a: ω2 = 2.70361 and M8: ω = 2.46384 that correspond to significant BEB site findings. The branch site models also have high foreground branch ω ratios f: ω2a = 723.98940, ω2b = 723.98940 for branch 1 consisting of *Arabidopsis*, *Beta* and *Brassica* species. *Nelumbo* branch site also indicates evolutionary selection pressure f: ω2a = 1.01816, ω2b = 1.01816 but not as strongly as branch 1 (supplementary dataset 2).

### Divergence estimates for selected gene candidates

DIVERGE tool^46^ v3.0 analyses gene tree clusters; host versus non-hosts for estimating the type of divergence observed in the comparative sets. Type I and Type II functional divergence ratios for the protein sequences were assessed via the LRTθ and θ-II values, respectively. Supplementary Table S5 summarizes the evolutionary pressure exerted on the protein residues. Type I divergence signifies amino acid patterns that are conserved in one clade but are variable in the other that indicates varying evolutionary rate hence, functional constraints. Such sites are expected to have experienced shifted functional constraints and are regarded important for functional divergence. Type I divergence, confirmed by LRT θ values, was found in 5 known and 2 transcriptome analysis-based candidate genes namely; LYK12 (0.351), DELLA (0.909), HMGR-CoA (5.330), CCaMK (6.723), SYMRK (17.239), AAA-ATPase (0.448), and *β*-expansin (3.138). No type II divergence was identified. The *p* value(s) for LRR receptor-like S/T kinase and 6-phosphogluconate dehydrogenase were > 0.05. Hence, these proteins were not considered for divergence classification.

The results from in silico phylogenetic analyses can be summarized as follows. Seventeen out of the twenty-two selected gene targets were analysed for extent of influence on structure and function with respect to evolutionary constraints such as selection pressure. Seven gene targets belonged to set 1 (putative novel candidate genes), eight targets belonged to set 2 (known candidate genes) and two gene targets (*CCD7* and *HMGR-CoA*) overlapped in both sets. These were divided into three groups based on in silico phylogenetic analyses and the extent to which they experienced selection pressure namely, high, moderate and low evolutionary constraints. An elaborate account on significance of these three groups with respect to importance in modulating AM symbiosis is presented in the “Discussion” section.

## Discussion

Phylogenetics coupled with either transcriptomics/genomics^28,29^ or reverse genetics^18^ has enabled identification of core genes crucial for AM symbiosis in host plants. Many studies have linked the evolutionary pressures exerted on AM symbiosis with mutualism sustenance in hosts and loss of symbiotic ability (mutualism desertion) or transition to weaker associations in other plant species^9,11,52,53^. This study proposed that the function of AM symbiosis candidate genes and their encoded proteins, that are present in both host and non-hosts of AM fungi, might be influenced by the rate of evolution and environmental constraints towards enabling or rejecting AM colonization.

Comparative transcriptome analysis of host and “surrogate non-host” cultivars of tomato were used to determine the extent of mycorrhization-specific gene regulation in closely-related species. The non-cultivar tomato cv. Grafter is also known to have better yield and higher innate resistance to root borne pathogens such as *Verticillium* sp. and *Fusarium* sp^31^. The DEGs were then tested for their involvement in AM-plant interaction across model host and non-host families via an extensive phylogenetic study.

The AM symbiosis-specific cluster obtained from comparative transcriptomics of host and non-host tomato cultivars were sorted for DEGS with most significant *p* value, excluding the uncharacterized protein. The 100 top hits (*p* value = 0.00005, − 2 ≤ log fold change ≥ 2) out of these 963 genes were either known candidates or putative novel candidate genes. Out of these DEG top hits, 10 gene targets were randomly picked from the same pathway as the “set 1 genes” discussed in this study to avoid bias for qRT-PCR validation of RNA seq results. The qRT-PCR expression fold changes were partially replicated wherein, 3 out of 10 targets selected for qRT-PCR validation showed high correlation with RNA seq data (r^2^ = 0.99), namely Gluc 4_66, Phe 12_14 and Suc 9_67. Remaining gene targets did not show statistically significant fold changes between mycorrhized and non-mycorrhized host. However, the qRT-PCR genes showed similar expression pattern (up- and down- regulation) as that of the RNA seq genes. It may be noted that the contributing pathways (common with “set 1 genes”), from which the genes for qRT-PCR are picked, have biological significance. The biological significance of the pathways of genes that lacked significant fold change in expression in qRT-PCR is validated via gene sets discussed in the phylogenetic analysis.

The multi-gene phylogenetic analysis included two sets of genes: set 1 comprised of 10 most significant differentially expressed AM-specific gene candidates obtained from the comparative transcriptome analysis termed as “putative gene candidates” and set 2 comprised of 12 “known gene candidates”. These genes could be placed/identified under three groups depending upon the evolutionary constraint and structural changes experienced by the orthologous genes in different AM-host and non-host species.

Group 1 consisted of genes showing high evolutionary constraints, which may influence the ability of plant species to become an AM-host. This group comprised of only two known candidate genes; *SYMRK*^54,55^ and *CCaMK*^56,57^. These genes showed significant differences in amino acid composition of the analysed protein sequences in a host- and non-hosts specific manner in all the in silico analyses including MEME, CODEML and DIVERGE. SYMRK showed amino acid changes specific to the four non-hosts selected in predicted motifs in MEME analysis. Such amino acid change could contribute to functional differences that affect AM symbiotic interactions between host and non-hosts; the substitution of proline (P) to glutamate (E) is interesting since glutamate is a negatively charged hydrophilic amino acid known to be involved in signalling and equilibrating protein structure^58^. It has also been known to affect protein interactions via post-translational modifications^58^. Similarly the substitution of phenylalanine (F) to tyrosine (Y) in all four non-hosts might require investigation into the role of tyrosine in “molecular recognition” that affects protein interactions^59^. The site and branch site CODEML models for SYMRK indicates the involvement of adaptive evolution with the change in amino acid pattern specific to non-host while presence of type 1 divergence confirms that its protein residues are under selection pressure amongst paralogous clades subject to gene duplication. These amino acid sequence changes might influence protein function, which in turn may further modulate interaction of host with AM fungi compared to non-hosts species.

In the same way, CCaMK also showed positional as well as amino acid sequence changes in predicted motifs via MEME in at least 3 non-hosts species (excluding *Nelumbo*). Amino acid substitution to valine (V) and serine (S) respectively might affect the protein signalling. Valine is a branched chain amino acid (BCAA) that is known to be involved in signalling pathways^60^ but not well explored in plant–microbe signalling. Similarly, serine-rich repeat proteins (SRRPs) have been evaluated and confirmed for their role in assisting microbe-host interaction^61^ but are not yet discovered for their role in plant-AM symbiotic signalling. The *CCaMK* CODEML site and branch-site model supports variable selection that favours non- synonymous mutation while presence of type I divergence suggests that the amino acid sequences show structural diversity that might affect their functional roles due to difference in evolutionary rates^46^. *SYMRK* and *CCaMK* are part of the Common Symbiotic Signalling Pathway (CSSP), and are reported to be involved in both rhizobial and AM symbiotic interactions^19,62^.

Group 2 consisted of genes that exhibited moderate evolutionary constraints, which may modulate AM-symbiosis outcome. In our analysis, this group comprises of genes that could not fit into one of the in silico test models (MEME/CODEML/DIVERGE) applied to trace evolutionary changes. This includes *DELLA*, *HMGR-CoA*, *LYK12*, *LYK10*, *AAA-ATPase*, *ß-expansin*, and *LRR receptor-like Ser/Thr protein kinase*. *DELLA* and *AAA-ATPase* did not show any significant branch-site model deviations in CODEML module, *LYK10* did not show divergence, while the rest did not have a non-host specific amino acid changes in MEME output. Nonetheless, the results do confirm that they have been affected by speciation and environmental factors by confirming presence of type I divergence.

DELLA shows amino acid substitution of leucine (L) and isoleucine (I) to methionine (M) specific to the non-host species. While this might seem to be a no-charge substitution that would not affect the property or functionality of DELLA protein, the sulfur-containing methionine has been proven to have unique properties that enables “antioxidant defence” and is also involved in stabilizing protein structure^63^. Further, even though the hydropathy does not change significantly (hydrophobic (L and I) to moderate (M)), the solubility measure changes from ~ 2 (L) and ~ 3 (I) to ~ 5 (M). The DELLA site models of CODEML further support the theory of adaptive evolution via positive selection. *DELLA* has been previously found to be involved in antagonism of GA signalling that regulates extent of AM symbiosis^16,64^.

Likewise, the candidate gene *AAA-ATPase At4g25835-like*, is known to be involved in protein degradation^65^ but has not been extensively studied with respect to AM symbiosis^66,67^ hence, can be novel candidates for exploration of their importance in the plant-mutualist interaction. The shift in AAA-ATPase motifs from uncharged amino acids valine (V), serine (S) and alanine (A) to negatively charged glutamic acid (E) for non-host proteomes (excluding *Nelumbo*) can have implications as discussed for SYMRK^58^. It also represents a change in adjacent amino acid sequence from arginine (R) and cysteine (C) to lysine (K) wherein R and K are both positively charged hydrophilic amino acids. Hence, this might also be attributed to properties other than the obvious such as solubility or the pKa. Presence of variable evolutionary constraints on *AAA-ATPases* was confirmed by the CODEML site models and presence of type 1 divergence.

For HMGR-CoA, the MEME output showed change in amino acid from aspartate (D) to glutamic acid (E) in non-hosts (excluding *Nelumbo*) that again corresponds to properties discussed for SYMRK glutamate substitution^58^. The CODEML site and branch-site models also had a very high positive selection ratio that confirms presence of non-synonymous mutation and divergence outputs supported the hypothesis of evolutionary constraints acting on these protein residues in paralogous clades. *SYMRK* works with *HMGR-CoA* to transfer perception signals from pre-contact stages of plant-AM interactions till contact to initiate calcium spiking via *CCaMK*^19^. It is interesting to note that *SYMRK* and *CCaMK* showed high evolutionary constraints (group 1), while *HMGR-CoA* was categorized for medium evolutionary constraints (group 2) between hosts and non-hosts.

*LYK12*, *ß-expansin* and *LRR receptor-like Ser/Thr protein kinase* does not show non-host cluster specific MEME changes. However, their CODEML and DIVERGE output support the presence of strong selection pressure acting on the respective proteins. Further, *ß-expansin* is known to enable arbuscule accommodation in plant cells and is up-regulated during AM symbiosis^68^. While its roles in hosts species (tomato and *Medicago*) has been established, this study expands a scope for this gene to be tested for functional deviation in non-host plant species that might cause defects in establishing mycorrhizal symbiosis and would be a useful area for further research^68^.

The possibility that selection pressure drives the differences in AM susceptibility of hosts and non-hosts at early stages are further supported by the CODEML and divergence results of *LYK12* and *LRR receptor-like Ser/Thr protein kinase*. *LYK12* is a LysM domain receptor required for AM colonization of hosts^69^ while *LRR receptor-like Ser/Thr protein kinase* is an LRR receptor-like serine/threonine-protein kinase possibly involved in plant- pathogen interactions^70–72^. There is a lot of potential to identify the components of this gene enabling AM symbiosis in hosts versus non-hosts that might lead to further regulators involved in the associated pathways. A recent study has also depicted induction of pathogen-related signalling cascade in *Arabidopsis* during later stages of host-driven AM colonization by *R. irregularis*^17^.

Group 3 consisted of genes that exhibited low evolutionary constraints. These genes fell short of demonstrating non-host specific changes in any two or all categories (MEME/ CODEML/ DIVERGE) amongst the applied tools used to trace evolutionary changes. This group comprises of the transcriptome data candidate *6-phosphogluconate dehydrogenase* that did not have any non-host specific amino acid changes in MEME. Although, the CODEML site models strongly support positive selection while the branch-site model also indicate that the protein residues experience evolutionary constraints, there is only a marginal presence of type I divergence (LRTθ = 0.033) that does not adhere to the *p* ≤ 0.05 cut off hence, could not be considered. The candidate gene *6-phosphogluconate dehydrogenase* has been recently known to be a part of the plastid proteome induced during AM symbiotic interactions^73^ and has considerable scope to be explored for its role in supporting the plant-AM mutualism. This group include *6-phosphogluconate dehydrogenase, POLLUX*, *CCD8*, *CCD7*, *CNGC15*, *calcium binding protein (CML45-like), scarecrow-like protein* and *S-adenosyl-L-methionine*. In our phylogenetic analysis we could not identify evolutionary constraints that might influence structure and function of these proteins to distinguish between hosts and non-hosts. However, these genes have been previously reported to play crucial functions in plant-AM interactions^17,19,74,75^. Such non-coherence in our observation provides scope for carrying out an independent replication study by including larger number of hosts and non-hosts species.

## Conclusion

The outcomes of the research presented here suggests that the approach followed is a valuable guide for further exploration of molecular basis of AM symbioses in hosts and non-hosts and how evolutionary constraints affect the gene function towards regulating mutualistic associations. These changes might equally be important determinants of symbiotic success versus failure in hosts and non-hosts respectively. It would be very interesting to undertake further structural studies to find out how the shift in amino acid composition of contributing motifs (MEME) and evolutionary pressures (CODEML and DIVERGE) might affect the nature and structure of these symbiosis-related proteins.

## Supplementary Information

Supplementary Information 1.

Supplementary Information 2.

Supplementary Information 3.
