# Supplementary material for: Important innate differences in determining symbiotic responsiveness in host and non-hosts of arbuscular mycorrhiza
Source: Sci Rep. 2021 Jul 14;11:14444. doi: 10.1038/s41598-021-93626-6 (PMC8280126; doi:10.1038/s41598-021-93626-6)
Supplement: Supplementary file 1 — Supplementary Information 1. [file 41598_2021_93626_MOESM1_ESM.docx]

**Important innate differences in determining symbiotic responsiveness in host and non-hosts of arbuscular mycorrhiza**

**Authors: Shalini Vasan^1,2^, Divya Srivastava^1^, David Cahill^2^, Pushplata Prasad Singh^1^*, Alok Adholeya^1^***

*Co-corresponding authors; email address: [adholeya1@gmail.com](mailto:adholeya1@gmail.com) and [pushplata.singh@teri.res.in](mailto:pushplata.singh@teri.res.in)

^1^TERI-Deakin Nanobiotechnology Centre, Sustainable Agriculture Division, The Energy and Resources Institute (TERI), Gurugram, Haryana, India

^2^School of Life and Environmental Sciences, Deakin University, Waurn Ponds Campus, Geelong, Victoria, Australia

**Method S1. Screening of host and non-host cultivars**

All the selected root cultures were inoculated with *Rhizophagus irregularis* DAOM 197198 spores (CMCC-ROC7) kindly provided by TERI’s Centre for Mycorrhizal Culture Collection (CMCC). Each culture plate comprised of 2-3 root segments (~7 cm in length) inoculated with 50 AM fungal spore and each batch comprised of 5 replicates for each culture line. This experiment was repeated thrice to confirm reproducibility of results. Ten weeks post inoculation, root sections were randomly picked from these AM inoculated root culture plates and residual media traces were de-ionized in citrate buffer (pH=6)^S[1](#_Doner,_L._W.)^ at 30°C ensuring the segments were not broken or damaged. These root segments were washed with distilled water followed by potassium hydroxide (KOH) wash and ink-vinegar staining to visualize AM colonization^S^[^2^](#_Vierheilig,_H.,_Coughlan,). Minor modifications included using 5% KOH and 3% ink-vinegar (prepared in 3% acetic acid) for staining. De-staining was accomplished using 5% acetic acid.

Colonization percentages were calculated for stained root segments after mounting them in lactoglycerol. Thirty root segments (~1 cm) were selected for each root culture line and each root segment was divided into four sections for calculating percentage of colonization^S^[^3^](#_Biermann,_B._&). The marker of successful symbiosis was either a well-developed hyphal network only, hyphal network and arbuscules and/or hyphae, arbuscules and vesicles. The sum total of percentage colonization assigned, was divided by the total number of sections (30*4 = 120) to deduce an average colonization percentage for the host lines.

**Method S2. RNA seq analysis.**

Reference based RNA-sequencing for all samples was conducted by a commercial company (Agrigenome Labs Pvt Ltd., Kerala, India). All RNA samples had a RIN value (RNA integrity Number) >9.0, that confirms suitability of these samples for downstream processing. Briefly, for transcriptome analysis of all root cultures, the rRNA transcripts were removed using RiboZero (Illumina, USA). Approximately 5µg of total RNA was used to prepare each RNA seq library using the TruSeq RNA sample prep kits (Illumina, USA) as per the manufacturer’s instructions. Poly-A containing mRNA molecules were purified using poly-T oligo attached magnetic beads. Following purification, the mRNA was fragmented into small pieces using divalent cations under elevated temperature. The cleaved RNA fragments were used to synthesize first strand cDNA via reverse transcriptase and random primers followed by second strand cDNA synthesis using DNA Polymerase I and RNaseH. The cDNA fragments were then subjected to an end repair process that included addition of a single ‘A’ base followed by ligation of the adapters. The products were purified and enriched via PCR to create the final cDNA library. Bioanalyzer plots were used at every step to assess mRNA quality, enrichment success, fragmentation sizes, and final library sizes. The size distribution of the sequencing library was determined by gel electrophoresis. Both pico-green and qPCR were used for library quantification prior to sequencing. This was followed by paired end run on Illumina’s HiSeq 2000/2500 platform to obtain 2×100bp reads to generate 60 million reads per sample as fastq files.

**Method S3. Quality control of sequenced data.**

Reads generated for each sample were quality tested based on the following parameters; base quality score distribution, sequence quality score distribution, average base content per read, GC distribution in the reads, PCR amplification issue (if any), over-represented sequences, checking for biasing of kmers and read-length distribution. Pre-processing of raw reads included trimming of the adaptor sequences and low-quality bases (quality cut off Q30) using AdapterRemoval-v2 (version 2.2.0^S^[^4^](#_Schubert,_M.,_Lindgreen,)). Ribosomal RNA sequences were removed by aligning the reads to silva database using bowtie2 (version 2.2.9^S^[^5^](#_Langmead,_B._&)) and subsequent workﬂow using samtools (version 1.3.1^S^[^6^](#_Li,_H._et)), sambamba (version 0.6.5^S^[^7^](#_Tarasov,_A.,_Vilella,)), BamUtil (version 1.0.13^S^[^8^](#_Jun,_G.,_Wing,)) tools and Agrigenome’s in-house scripts. It was necessary to ensure that the genes being analysed for differential expression studies are truly of plant origin instead of fungal (possible in the case of mycorrhized host tomato culture line, RomaVC2_Gi). Hence, the pre-processed and rRNA removed reads of RomaVC2_Gi samples were aligned with *Rhizophagus irregularis* isolate DAOM 181602=DAOM 197198 from Ensembl Fungi. The unaligned reads from this output, and pre-processed and rRNA removed reads of tomato Grafter non-mycorrhized and tomato Roma non-mycorrhized samples were aligned to the *S. lycopersicum* genome. The gene model was downloaded from Ensembl-Plants ({[ftp://ftp.ensemblgenomes.org/pub/plants/release41/
fasta/solanumlycopersicum/dna/Solanum_lycopersicum.SL2.50.dna.toplevel.fa.gz](ftp://ftp.ensemblgenomes.org/pub/plants/release41/fasta/solanumlycopersicum/dna/Solanum_lycopersicum.SL2.50.dna.toplevel.fa.gz)})and the alignment was performed using STAR program (version 2.5.3a^S^[^9^](#_Dobin,_A._et)). The reads aligned to the reference genome, were used for estimating expression of the genes and transcripts using cufflinks program (version 2.2.1). Differential gene expression analysis was performed using cuffdiff program of the cufflinks package^S^[^10^](#_Trapnell,_C._et)[^-^](#_Trapnell,_C._et_1)^S^[^13^](#_Roberts,_A.,_Trapnell,).

**Method S4. qRT-PCR validation of RNA seq gene expression changes.**

The reverse transcription was done using iScript cDNA Synthesis kit (BioRAD, California, USA) as per the manufacturer’s instructions with three biological replicates for each sample. Gene expression studies were carried out on the CFX-96 TouchTM Real- time PCR detection system (Bio-Rad Labs, Inc.). Each reaction was had a total volume of 20 μl that included 0.5 μM primers, 3 μl diluted cDNA (50 ng/μL), and 10 μl 2× SYBR Green PCR Master Mix (Bio-Rad Labs, Inc.). The qRT-PCR program included initial denaturation at 95°C for 30 secs, 39 cycles of denaturation 95°C for 10 secs and annealing at T_a_ for 30 secs, melt curve from 65°C to 95°C with increments of 0.5°C after every plate reading step.

Ten gene targets were randomly selected from the differentially expressed (DEG) gene-sets of RNA seq data at FDR ≤ 0.005. This enabled ruling out any bias while validating the transcriptomics data. All genes were part of the top 15 metabolic pathways regulated in response to AM inoculation as described in figure S3. Two genes (Phosphoglycerate kinase (PGK) and Elongation factor 1α (EF1a)) that are commonly used as references in Solanum lycopersicum^S^[^14^](#_Fuentes,_A._et) were used as reference genes. Relative gene expression levels of each gene were calculated using the 2^−△△^ Ct method. Gene-specific primers were showed in Supplementary Dataset 3.

**Method S5.** **Confirmation of the status of gene targets in host and non-host proteomes.**

Both nucleotide and protein gene sequences for set 1 was obtained from the Refseq database BLAST results while for set 2 fasta sequences were downloaded from NCBI database (<https://www.ncbi.nlm.nih.gov>). *Lotus japonicus* and *Beta vulgaris* did not have a published proteome available in NCBI. Hence, gene sequences for these were obtained via the gene prediction tool, Augustus version 2.5.5^S^<15> (<http://bioinf.uni-greifswald.de/augustus/>).

Two gene targets overlapped in set 1 and 2, *Carotenoid cleavage dioxygenase7* (*CCD7*) and *3-hydroxy-3-methyl-glutaryl-CoA reductase (HMGR-CoA*) and were amongst the marker genes that validated the robustness of the RNA seq parameters used to evaluate differentially expressed genes (DEGs).

**Method S6. Identifying conserved regions for functional analysis** Selected gene sets were subjected to offline BLAST against the plant proteomes and the resultant accession IDs were used for Conserved Domain Database (CDD) analysis^S^<16> (<https://www.ncbi.nlm.nih.gov/cdd/>) to confirm presence or absence of functional domains in the selected species. Sequences that were identified through homology analysis by BLAST^S^[^17^](#_Altschul,_S._F.,) were confirmed for functional similarity through CDD analysis, which identifiedthe presence of conserved domains within a protein sequence. Only genes that were present in at least 10 of the 11 selected genomes were included in further phylogenomic analysis. For analysis of evolutionary genetic patterns for each of the selected genes, fasta sequences of homologous genes among the host and non-host species were used as input for Multiple Sequence Alignment (MSA) via MUltiple Sequence Comparison by Log-Expectation (MUSCLE), an EMBL_EBI tool ([https://www.ebi.ac.uk/
Tools/msa/muscle/](https://www.ebi.ac.uk/Tools/msa/muscle/))^S^[^18^](#_Madeira,_F._et). The output was obtained in Pearson/fasta format. The aligned protein files were concatenated to consist of only the conserved regions using Gblocks 0.91b tool (<http://phylogeny.lirmm.fr/phylocgi/onetask.cgi?tasktype=gblocks>). The aligned and concatenated protein files were used for building a gene tree via MEGAX, motif prediction via MEME and DIVERGE analysis. Another set of aligned and concatenated conserved sequence file was prepared with the gene nucleotide sequences to be used as input for CODEML analysis.

**Table S1.** Details of host and non-hosts used for comparative transcriptomics and their respective treatments.

| **Root culture codes** | **Line details** |
| --- | --- |
| RomaVC2_Gi | Mycorrhized host line: roots of tomato RomaVC2 inoculated with 50 *R. irregularis* spores and harvested at 22^nd^ day. |
| RomaNMyc | Host root line sub-cultured and allowed to grow for 21 days without any mycorrhizal inoculation hence, non-mycorrhized (NMyc). Harvested at 22^nd^ day. |
| GrafterNMyc | Non-host root line sub-cultured and allowed to grow for 21 days on M media without any mycorrhizal inoculation (NMyc). Harvested at 22^nd^ day. |

**Table S2.** Hosts and non-host genomes selected for the phylogenetics study^S^[^19^](#_Bravo,_A.,_York,).

| **Plant species** | **Type of plant** | **Status (host/non-host)** | **Family** |
| --- | --- | --- | --- |
| *Solanum lycopersicum* | Dicot | Host | Solanaceae |
| *Solanum tuberosum* | Dicot | Host | Solanaceae |
| *Lotus japonicus* | Dicot | Host | Fabaceae |
| *Medicago truncatula* | Dicot | Host | Fabaceae |
| *Glycine max* | Dicot | Host | Fabaceae |
| *Oryza sativa* | Monocot | Host | Poaceae |
| *Zea mays* | Monocot | Host | Poaceae |
| *Beta vulgaris* | Dicot | Non-host | Amaranthaceae |
| *Nelumbo nucifera* | Dicot | Non-host | Nelumbonaceae |
| *Brassica rapa* | Dicot | Non-host | Brassicaceae |
| *Arabidopsis thaliana* | Dicot | Non-host | Brassicaceae |


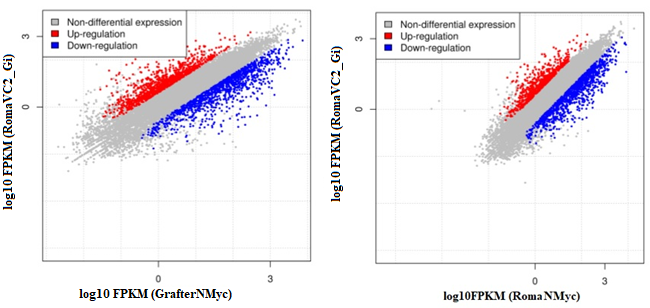


**b**

**a**

## Figure S1. FPKM (fragments per kilobase, per million) scatter plots representing differential gene expression status between non-mycorrhized host/ non-host and mycorrhized host samples. a) comparison of gene expression status between non-inoculated non-host (GrafterNMyc) and mycorrhiza-inoculated host (RomaVC2_Gi); b) comparison of gene expression status between non-inoculated host (RomaNMyc) and RomaVC2_Gi. The expression values closer to diagonal line marked in grey area represent neutral or similar expression. Red scatter points represent genes up-regulated in RomaVC2_Gi while blue area represents the down-regulated gene sets in RomaVC2_Gi compared to GrafterNMyc and RomaNMyc respectively.

**
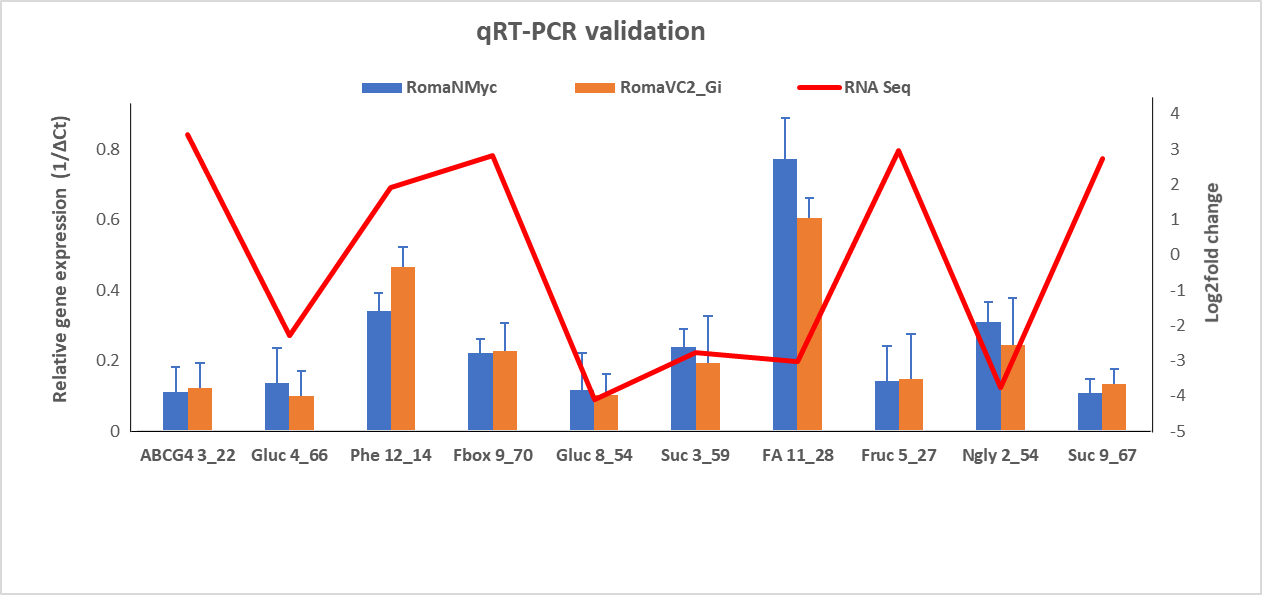
**

**Figure S2.** Validation of RNA seq data via qRT-PCR. Gene expression levels (1/ΔCt) were compared between non-mycorrhized host, RomaNMyc (blue) and mycorrhized host, RomaVC2_Gi (orange). Normalized gene expression was calculated using the 2^-(ΔΔCt)^ method; r^2^ = 0.55. Secondary axis (red line) indicates the log2fold change in RomaVC2_Gi compared to RomaNMyc as observed in RNAseq data for this gene set. The gene codes used in the graph, log2fold change values, correlation with RNA seq gene expression status and statistical analysis outputs are detailed in Supplementary dataset 3. Data are averages ± S.E.M., n =9.


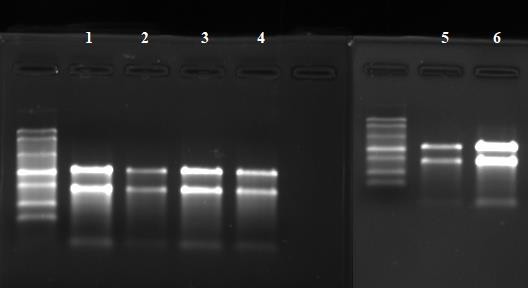


**Figure S3:** Gel electrophoresis of DNase treated RNA. RNA integrity was confirmed by the presence of 28S (~5 kB) and 18S (~1.8 kB) rRNA bands using the 2× ssRNA loading dye (NEB, USA). Absence of fluorescence near the sample wells indicate absence of higher molecular weight compounds (DNA/ proteins). Well 1 and 2 represent biological replicates of RomaNMyc, well 3 and 4 represent biological replicates of GrafterNMyc, and well 5 and 6 represent biological replicates of RomaVC2_Gi.


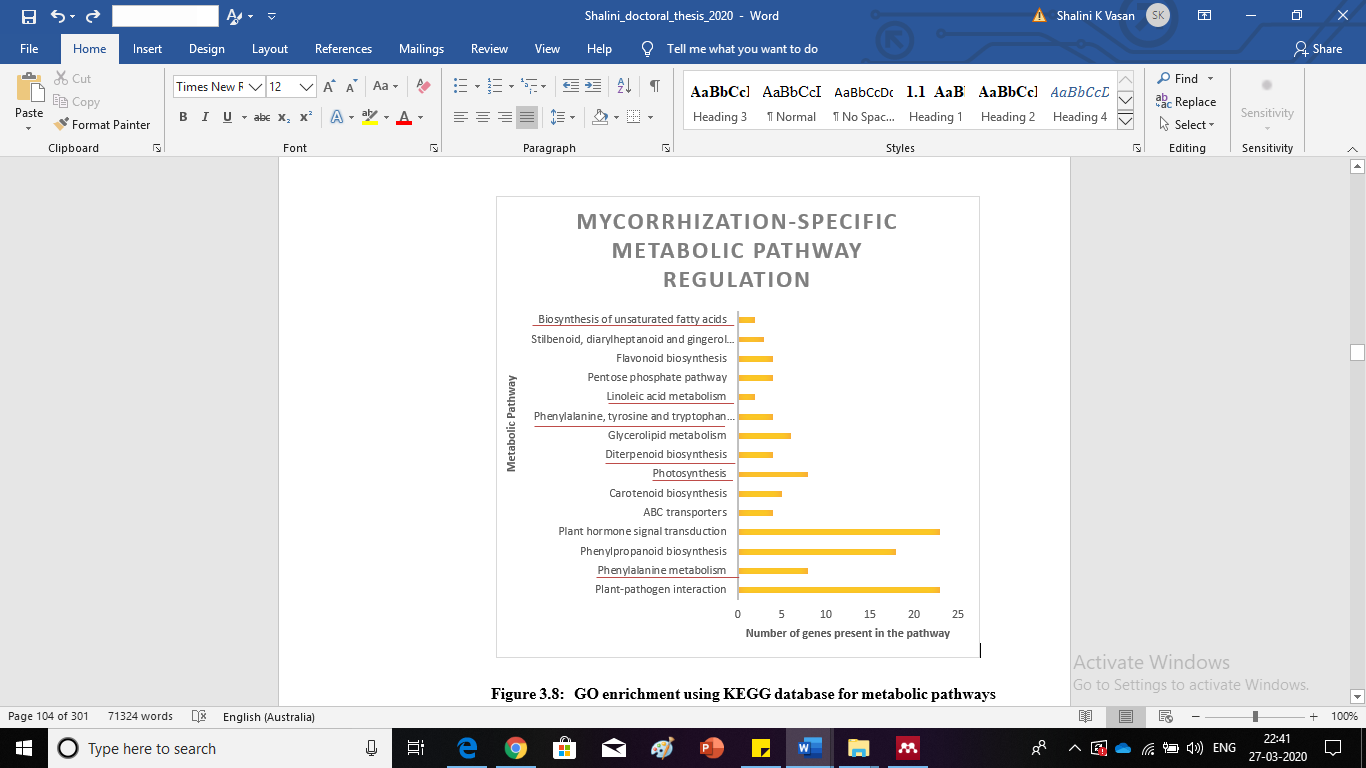


**Figure S4.** GO enrichment using KEGG (Kyoto Encyclopedia of Genes and Genomes) database for metabolic pathways responsive to AM symbiosis. This graph represents the metabolic pathways differentially regulated in response to inoculation of host with AM *R. irregularis*. 92 metabolic pathways were involved in the process and in order to enable visualization of results, top 15 pathways (based on *p* value) were functionally characterized for graphical representation using KEGG database via KOBAS^S^[^20^](#_Wu,_J.,_Mao,). KEGG is literally the encyclopedia of biological, cellular and molecular functions pertaining to a gene. KO (KEGG Orthology) is used to assign functional classification to the targeted genes^S^[^21^](#_Kanehisa,_M.,_Furumichi,).

**Table S3.** Status of set 1 (putative novel candidates) in host and non-host proteomes.

| **Protein details** | **Superfamily** (CDD) | **Status in host/ non-host** |
| --- | --- | --- |
| 6-phosphogluconate dehydrogenase,  decarboxylating2 | - GND superfamily - 6PGD superfamily | Present in all |
| Leucine-rich repeat (LRR) receptor-like-  serine/threonine-protein kinase | - Protein kinase (catalytic-domain) superfamily - TOMM system kinase protein superfamily | Present in all |
| Scarecrow-like protein 5 and protein 13 | - GRAS superfamily | Present in all |
| Putative calcium-binding protein CML45-like | - EF-hand superfamily (Ca2+ binding site) | Present in all |
| β-expansin | - Expansin-like B1(provisional) superfamily - DPBB-1 (double-psi beta-barrel fold) superfamily | Present in all |
| Pathogenesis-related genes transcriptional  activator (PTI6) | - AP2 superfamily (DNA binding site) | Present in all |
| AAA-ATPase At4g25835-like | - AAA assoc-superfamily (ATP binding site) - P-loop NTPase domain superfamily | Present in all |
| GATA transcription factor | - ZnF_GATA superfamily (Zinc binding site; and   DNA binding region) | Present in all |
| S-adenosyl-L-methionine (SAM); salicylic  acid carboxyl methyltransferase | - Methyltransf_7 superfamily (SAM dependent   carboxyl methyltransferase) | Present in all |
| Ankyrin repeat, PH and SEC7 domain  containing protein secG-like | - Ank_2 superfamily | Present in all except tomato  and maize; ank repeat sites  available in all proteomes |

**Table S4.** Status of set 2 (known candidates) in host and non-host proteomes.

| **Protein details** | **Superfamily** (CDD) | **Status in host/ non-host** |
| --- | --- | --- |
| CASTOR | - BK-channel superfamily | Present in all; *Nelumbo* *nucifera* aligns to POLLUX |
| CNGC 15 | - CAP superfamily | Present in all, 25% query coverage and 75% identity found in *Lotus* |
| CCaMK | - Pkinase superfamily - TOM_kin_cyc superfamily - EF hand 7 superfamily | Present in all, 30% identity in *Beta* *vulgaris*. However, the  annotation and domain matched. |
| CYCLOPS/IPD3 | - Ezra superfamily - SH3 and anchor superfamily - Cnn-IN superfamily | Present only in hosts |
| DELLA | - GRAS superfamily - DELLA superfamily | Present in all. In *Lotus*, DELLA aligns to RAM1: Query coverage of 66% and identity 41% but only GRAS domain was present |
| HMGR CoA | - HMG-CoA reductase superfamily | Present in all except *Lotus* |
| POLLUX | - BK-channel superfamily | Present in all |
| SYMRK | - STYKc superfamily - Pkinase_Tyr superfamily | Present in all |
| LYK10 | - PTK_Jak_rpt1 pseudokinase | Present in all |
| LYK12 | - TOMM_kin_cyc superfamily   (Protein kinase catalytic submit) | Present in all |
| CCD7 CCD8 | - Retinal pigment epithelial   membrane protein superfamily | Present in all |


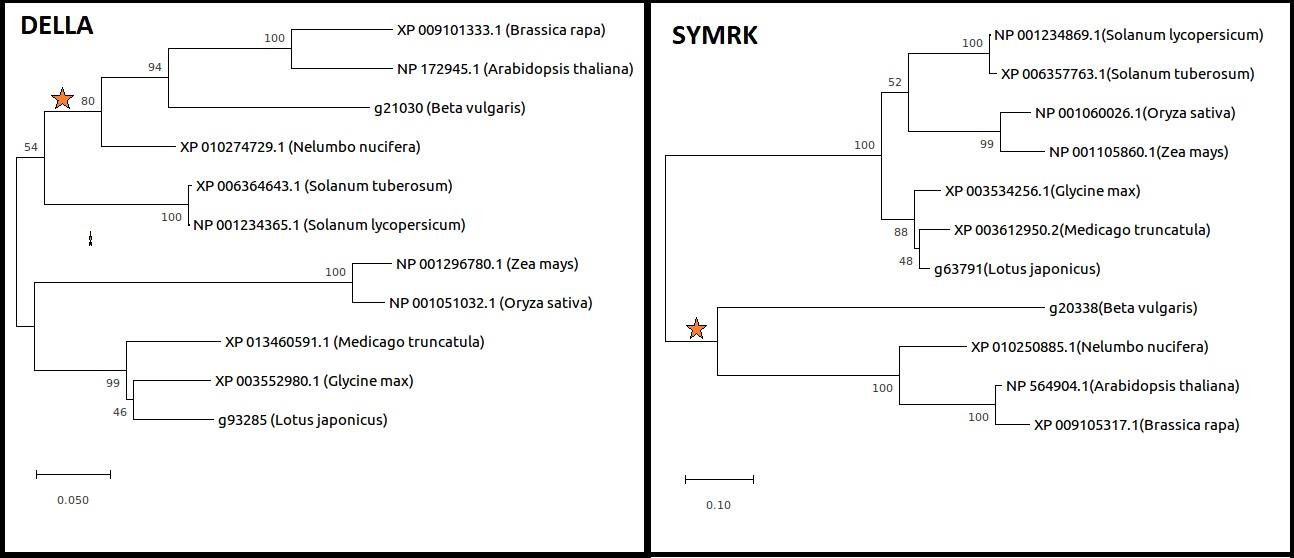


**Figure S5.** Representative gene trees for category 1. One evolutionary event (
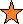
) marking the divergence of gene cluster between host and non-hosts. The gene trees belonging to this category completely match the species tree (one non-host gene cluster). Left image represents the gene tree for *DELLA* and right image represents tree for *SYMRK*. Non-host species include *N. nucifera, B. vulgaris, A. thaliana* and *B. rapa* while the remaining are hosts.


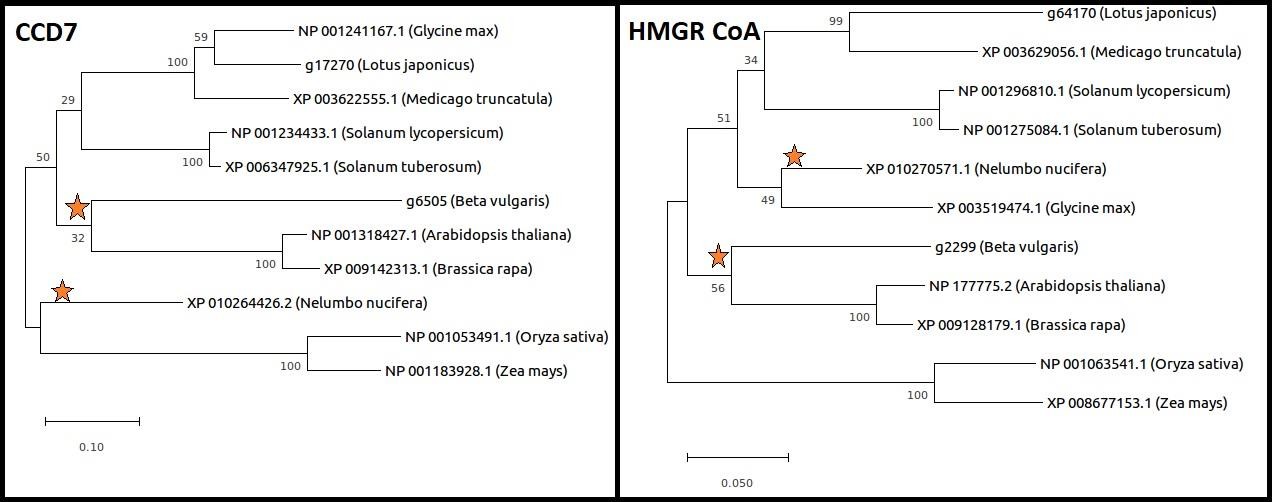


**Figure S6.** Representative gene trees for category 2. Two different evolutionary events (
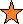
) marking the divergence of gene cluster in selected non-hosts. Left image represents the gene tree for *CCD7* and right image represents gene tree for *HMGR-CoA*.


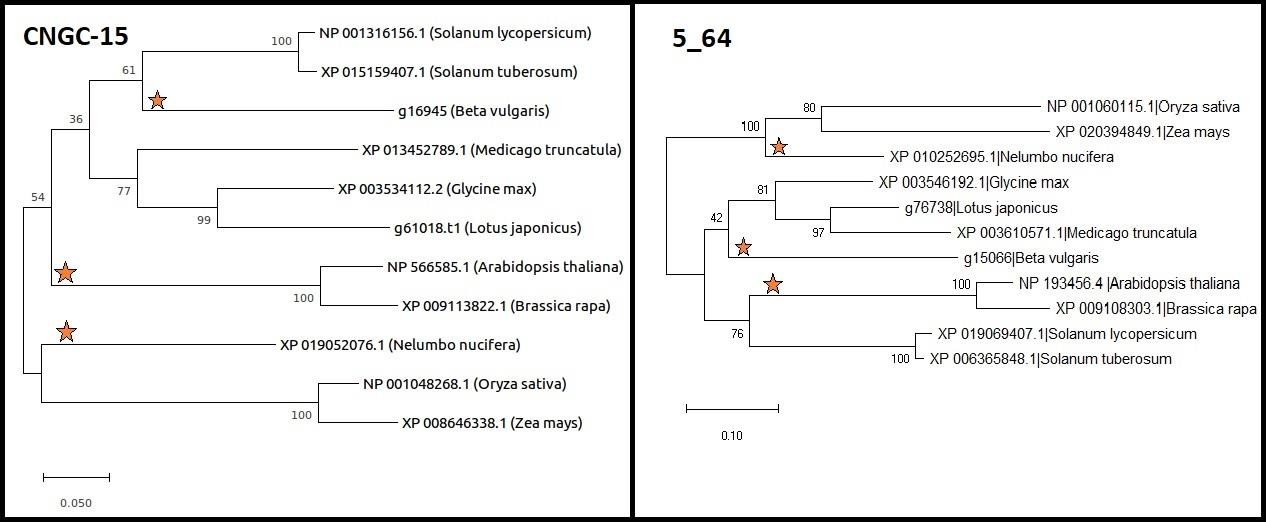


**Scarecrow-like protein**

**Figure S7.** Representative gene trees for category 3. Evolutionary pressure is not strong enough to segregate host and non-host gene clusters unlike category 1 and 2. Left image represents the gene tree for *CNGC-15* and right image represents gene tree for *Scarecrow-like protein*.


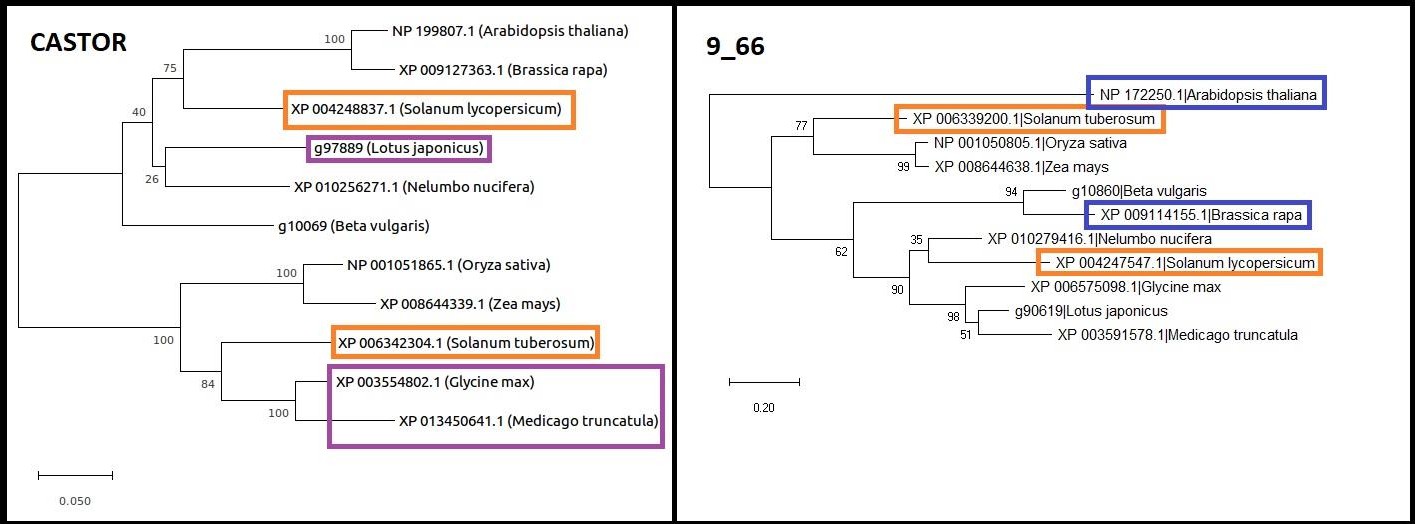


**Ankyrin repeat**

**Figure S8.** Representative gene trees for category 4. This figure represents genes that do not show clear classification of genes in selected species. Orange block represent species belonging to family Solanaceae but separated in the gene clusters. Similar pattern was observed in Fabaceae (purple box) and Brassicaceae (blue box).


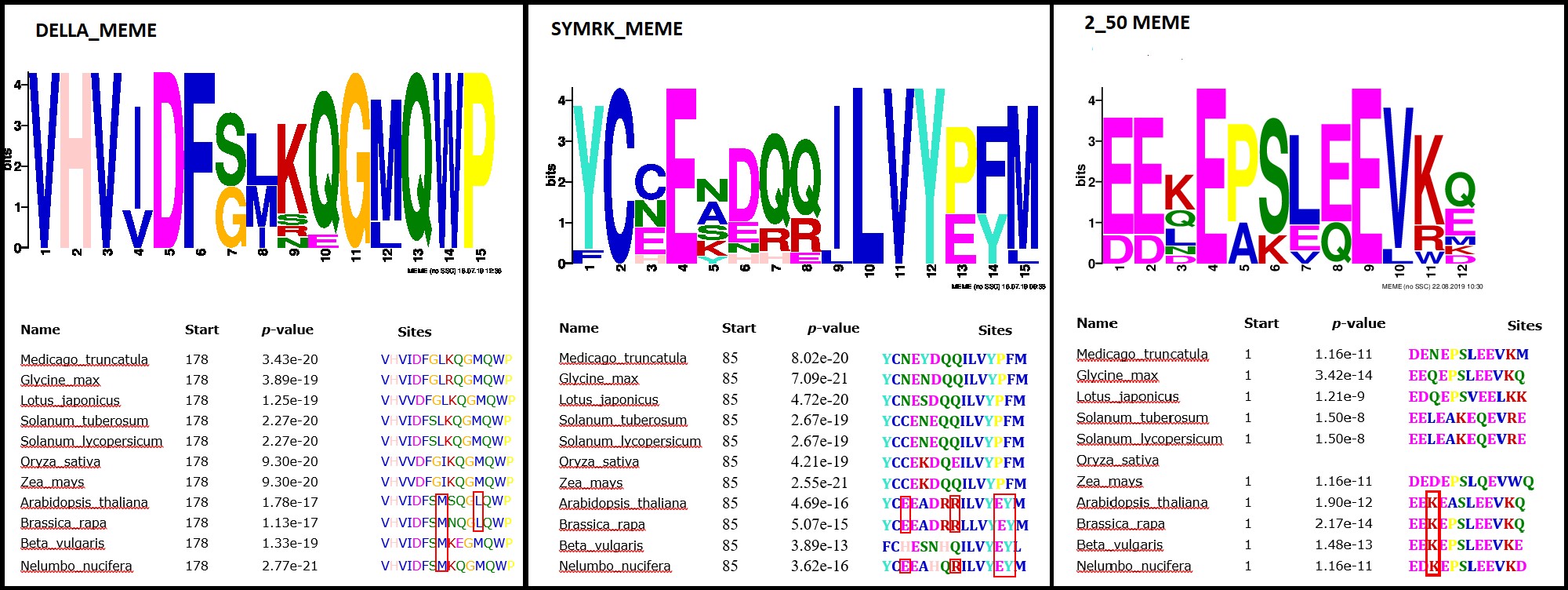


**CML45-like_MEME**

**Figure S9.** Amino acid changes in predicted motifs specific to 4 non-host genomes selected for the phylogenetics study. (Left to Right) MEME output for DELLA, SYMRK and calcium-binding protein (CML45-like) respectively. The non-hosts include *N. nucifera, B. vulgaris, A. thaliana* and *B. rapa* while the remaining are hosts. Red boxes represent amino acid changes in non-hosts for predicted motifs.


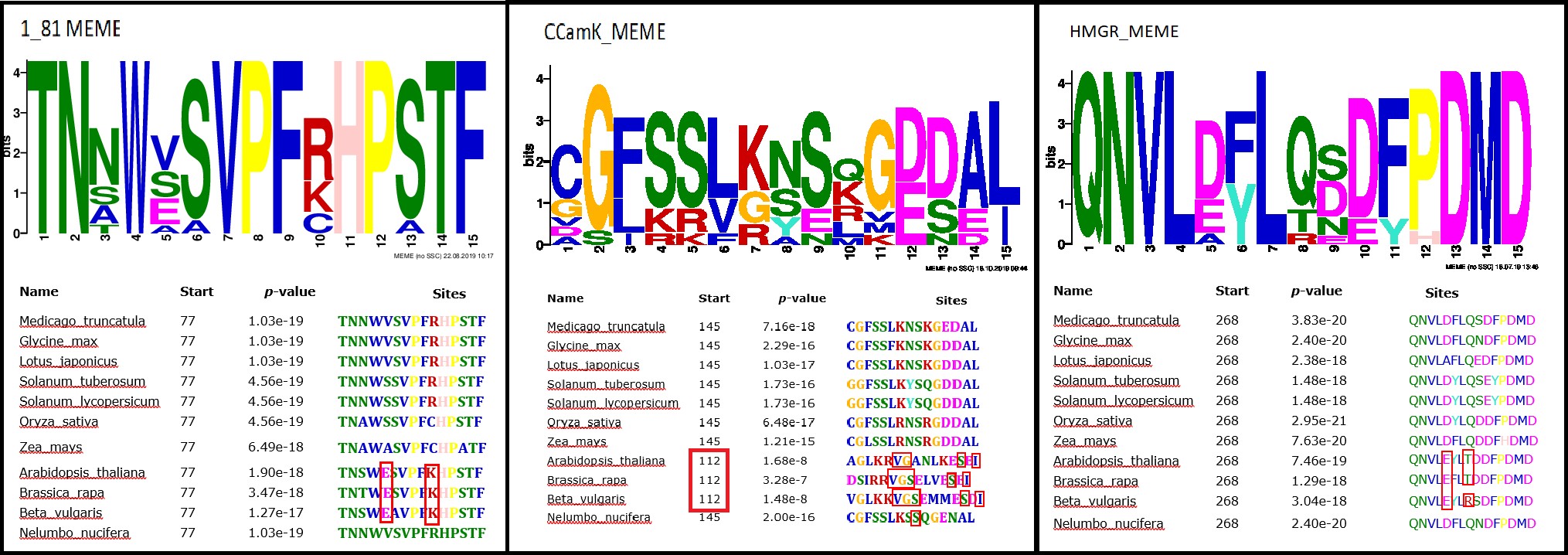


**HMGR-CoA_MEME**

**CCaMK_MEME**

**AAA-ATPase_MEME**

**Figure S10.** Amino acid differences found in 3 out of 4 selected non-hosts. (Left to Right) MEME output for AAA-ATPase, CCaMK and HMGR-CoA respectively. Red boxes represent amino acid changes in non-hosts for predicted motifs.


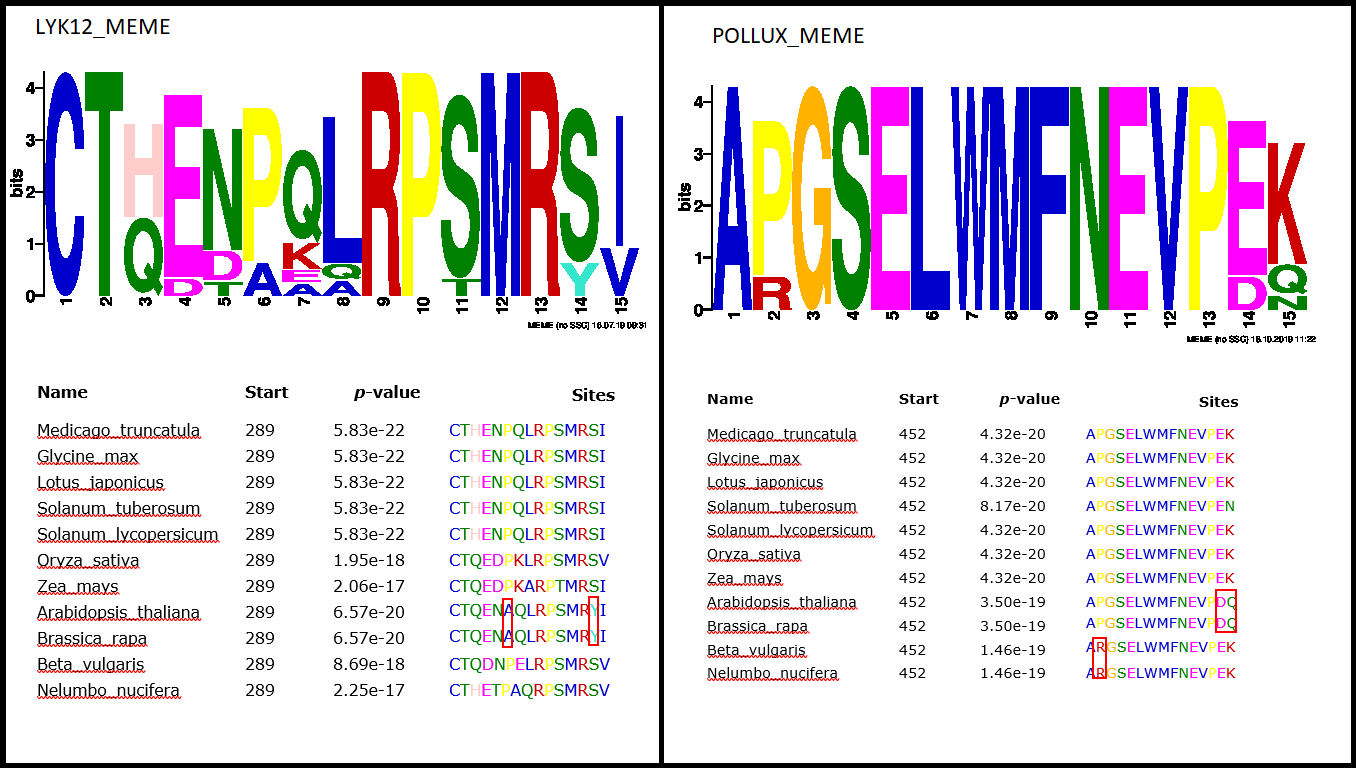


**Figure S11.** Family-specific motif differences. This figure represents amino acid sequence difference that were plant-family specific and cannot be correlated to any biological functional changes**.** (Left to Right) MEME output for LYK12 and POLLUX respectively. Red boxes represent amino acid changes in non-hosts for predicted motifs.

**Table S5.** Estimation of type-1 divergence in gene targets.

| **Type-1 Divergence** | **LYK12** | **DELLA** | **HMGR-**  **CoA** | **CCaMK** | **SYMRK** | **AAA- ATPase** | **β-expansin** | **LRR receptor-like ser/thr kinase** | **6-phospho**  **gluconate dehydrogenase** |
| --- | --- | --- | --- | --- | --- | --- | --- | --- | --- |
| **θML** | 0.0552 | 0.079764 | -7221.2 | 0.4632 | 0.5064 | 0.0656 | -0.348383 | -0.008337 | 0.0106 |
| **SE θ** | 0.093056 | 0.083633 | -0.08975 | 0.178639 | 0.121967 | 0.098009 | -0.196678 | -0.020751 | 0.058117 |
| **LRT θ** | 0.351879 | 0.909615 | 5.330364 | 6.723368 | 17.238516 | 0.448001 | 3.137652 | 0.161407 | 0.033266 |
| ***p* value** | 0.031 | 0.047 | 0.036 | 0.0002 | 0.007 | 0.024 | 0.00004 | 0.339 | 0.122 |
| **Sites** | 322 | 429 | 479 | 224 | 274 | 193 | 203 | 891 | 466 |

## References

### Doner, L. W. & Becard, G. Solubilization of gellan gels by chelation of cations. *Biotechnol. Tech.***5**, 25–28[, https://doi.org/10.1007/BF00152749](file:///C:\Users\Shalini\Desktop\shalini%20phd%20temp\publications\Research%20article%201\,%20https:\doi.org\10.1007\BF00152749) (1991).

### Vierheilig, H., Coughlan, A. P., Wyss, U. R. S. & Piche, Y. Ink and Vinegar, a Simple Staining Technique for Arbuscular-Mycorrhizal Fungi. Appl. Environ. Microbiol. 64, 5004–5007, <https://doi.org/10.1128/AEM.64.12.5004-5007.1998> (1998).

### Biermann, B. & Linderman, R. G. Quantifying vesicular-arbuscular mycorrhizae : a proposed method towards standardization*. *New Phytol.* 63–67, <https://doi.org/10.1111/j.1469-8137.1981.tb01690.x> (1981).

### Schubert, M., Lindgreen, S. & Orlando, L. AdapterRemoval v2: Rapid adapter trimming, identification, and read merging. *BMC Res. Notes***9**, 1–7, <https://doi.org/10.1186/s13104-016-1900-2> (2016).

### Langmead, B. & Salzberg, S. L. Fast gapped-read alignment with Bowtie 2. *Nat. Methods***9**, 357–359, <https://doi.org/10.1038/nmeth.1923> (2012).

### Li, H. et al. The Sequence Alignment/Map format and SAMtools. *Bioinformatics***25**, 2078–2079, <https://doi.org/10.1093/bioinformatics/btp352> (2009).

### Tarasov, A., Vilella, A. J., Cuppen, E., Nijman, I. J. & Prins, P. Sambamba: fast processing of NGS alignment formats. *Bioinformatics***31**, 2032–2034, [https://doi.org/10.1093/bioin formatics/btv098](https://doi.org/10.1093/bioinformatics/btv098) (2015).

### Jun, G., Wing, M. K., Abecasis, G. R. & Kang, H. M. An efficient and scalable analysis framework for variant extraction and refinement from population-scale DNA sequence data. *Genome Res.***25**, 918–925, <https://doi.org/10.1101/gr.176552.114> (2015).

### Dobin, A. et al. STAR: ultrafast universal RNA-seq aligner. *Bioinformatics***29**, 15–21, <https://doi.org/10.1093/bioinformatics/bts635> (2012).

### Trapnell, C. et al. Transcript assembly and quantification by RNA-Seq reveals unannotated transcripts and isoform switching during cell differentiation. *Nat. Biotechnol.***28**, 511–515, <https://doi.org/10.1038/nbt.1621> (2010).

### Trapnell, C. et al. Differential analysis of gene regulation at transcript resolution with RNA-seq. *Nat. Biotechnol.***31**, 46, [https://doi.org/10.1038/nbt.2450](https://doi.org/10.1038/nbt.2450%20) (2012).

### Roberts, A., Pimentel, H., Trapnell, C. & Pachter, L. Identification of novel transcripts in annotated genomes using RNA-Seq. *Bioinformatics***27**, 2325–2329, [https://doi.org/10. 1093/bioinformatics/btr355](https://doi.org/10.1093/bioinformatics/btr355) (2011).

### Roberts, A., Trapnell, C., Donaghey, J., Rinn, J. L. & Pachter, L. Improving RNA-Seq expression estimates by correcting for fragment bias*. Genome Biol.***12**, R22, [https://doi.org/10.1186/gb-2011-12-3-r22](https://doi.org/10.1186/gb-2011-12-3-r22%20) (2011).

### Fuentes, A. *et al.* Reference gene selection for quantitative real-time PCR in Solanum lycopersicum L. inoculated with the mycorrhizal fungus Rhizophagus irregularis. *Plant Physiol. Biochem.* **101**, 124–131, <https://doi.org/10.1016/j.plaphy.2016.01.022> (2016).

### Stanke, M. & Morgenstern, B. AUGUSTUS: A web server for gene prediction in eukaryotes that allows user-defined constraints. *Nucleic Acids Res.***33**, 465–467, <https://doi.org/10.1093/nar/gki458> (2005).

### Marchler-Bauer, A. et al. CDD/SPARCLE: Functional classification of proteins via subfamily domain architectures. *Nucleic Acids Res.***45**, D200–D203, [https://doi.org/10.1093/nar/gkw1129](https://doi.org/10.1093/nar/gkw1129%20) (2017).

### Altschul, S. F., Gish, W., Miller, W., Myers, E. W. & Lipman, D. J. Basic local alignment search tool. *J. Mol. Biol.***215**, 403–410, <https://doi.org/10.1016/S0022-2836(05)80360-2> (1990).

### Madeira, F. et al. The EMBL-EBI search and sequence analysis tools APIs in 2019. *Nucleic Acids Res.***47**, W636–W641, [https://doi.org/10.1093/nar/gkz268](https://doi.org/10.1093/nar/gkz268%20) (2019).

### Bravo, A., York, T., Pumplin, N., Mueller, L. A. & Harrison, M. J. Genes conserved for arbuscular mycorrhizal symbiosis identified through phylogenomics. *Nat. Plants***2**, 1–6, [https://doi.org/10.1038/nplants.2015.208](https://doi.org/10.1038/nplants.2015.208%20) (2016).

### Wu, J., Mao, X., Cai, T., Luo, J. & Wei, L. KOBAS server: A web-based platform for automated annotation and pathway identification. *Nucleic Acids Res.***34**, 720–724, <https://doi.org/10.1093/nar/gkl167> (2006)

### Kanehisa, M., Furumichi, M., Tanabe, M., Sato, Y., Ishiguro-Watanabe, M., and Tanabe, M. KEGG: integrating viruses and cellular organisms. *Nucleic Acids Res.* **49**, D545–D551, [https://doi.org/10.1093/nar/gkaa970](https://doi.org/10.1093/nar/gkw1092) (2021).
